# Surveillance system integration: reporting the results of a global multicountry survey

**DOI:** 10.1016/j.puhe.2024.03.004

**Published:** 2024-04-10

**Authors:** E.D. Carter, D.E. Stewart, E.E. Rees, J.E. Bezuidenhoudt, V. Ng, S. Lynes, J.C. Desenclos, T. Pyone, A.C.K. Lee

**Affiliations:** a US Centers for Disease Control and Prevention, USA; b UK Health Security Agency, UK; c Public Health Agency of Canada, Canada; d National Institute for Communicable Diseases, South Africa; e International Association of National Public Health Institutes, Belgium; f International Association of National Public Health Institutes & Santé publique France, France; g World Health Organization, Geneva, Switzerland; h UK Health Security Agency & The University of Sheffield, UK

**Keywords:** Surveillance, Communicable diseases, Communicable disease control, Integrated disease surveillance, Population health surveillance

## Abstract

**Objectives:**

Currently, there is no comprehensive picture of the global surveillance landscape. This survey examines the current state of surveillance systems, levels of integration, barriers and opportunities for the integration of surveillance systems at the country level, and the role of national public health institutes (NPHIs).

**Study design:**

This was a cross-sectional survey of NPHIs.

**Methods:**

A web-based survey questionnaire was disseminated to 110 NPHIs in 95 countries between July and August 2022. Data were descriptively analysed, stratified by World Health Organization region, World Bank Income Group, and self-reported Integrated Disease Surveillance (IDS) maturity status.

**Results:**

Sixty-five NPHIs responded. Systems exist to monitor notifiable diseases and vaccination coverage, but less so for private, pharmaceutical, and food safety sectors. While Ministries of Health usually lead surveillance, in many countries, NPHIs are also involved. Most countries report having partially developed IDS. Surveillance data are frequently inaccessible to the lead public health agency and seldomly integrated into a national public health surveillance system. Common challenges to establishing IDS include information technology system issues, financial constraints, data sharing and ownership limitations, workforce capacity gaps, and data availability.

**Conclusions:**

Public health surveillance systems across the globe, although built on similar principles, are at different levels of maturity but face similar developmental challenges. Leadership, ownership and governance, supporting legal mandates and regulations, as well as adherence to mandates, and enforcement of regulations are critical components of effective surveillance. In many countries, NPHIs play a significant role in integrated disease surveillance.

## Introduction

The COVID-19 pandemic revealed weaknesses in public health surveillance systems worldwide, emphasising the need for more robust systems to detect and rapidly respond to public health threats.^1^ Better integrated systems for the detection and response to emerging and novel pathogens across the One Health spectrum (people, animals, and the environment) has been emphasised as vital.^2^ This concept of integrated disease surveillance (IDS) and integrated disease surveillance and response (IDSR) was first proposed by the World Health Organization (WHO) African Regional Office (WHO AFRO) in response to large outbreaks in West Africa.^3^ Nsubuga et al. described IDS as ‘*a combination of active and passive systems using a single infrastructure that gathers information about multiple diseases or behaviors of interest*.’^4^ The concept of IDS has evolved, and recent discourse focuses on case accuracy, digitisation, transparency, and adequate finance to sustain effective surveillance systems in the future.^5^ Also highlighted is the role of National Public Health Institutes (NPHIs) in integrating multiple sources of surveillance data to better understand threats to public health for more effective response.

Currently, there is no comprehensive picture of the global surveillance landscape and how IDS is conceptualised and implemented at the country level.^6^ In this paper, we present survey results of 65 of the member institutions of the International Association of National Public Health Institutes (IANPHI), which represents 110 government agencies and NPHIs across 95 countries, with the aim to collectively build public health capacity and improve disease prevention and response around the world.^7^ We assess the current state of surveillance systems, their level of integration, and the role of NPHIs in supporting surveillance and identify barriers and opportunities for the integration of surveillance systems.

## Methods

IANPHI is an association comprising NPHIs with representation from senior leaders. IANPHI supports the strengthening of Public Health Institutes through peer-to-peer support and communities of shared practice.^7^ IANPHI also works with partners, including the WHO and regional Centres for Disease Control to support their missions in developing solutions for country context actions in public health through the establishment of essential public health functions. At the time we conducted our study, IANPHI had 110 members in 95 countries.^7^

The multicountry cross-sectional survey that we report here was developed by a multidisciplinary working group supported by expert advice from IANPHI members and the WHO Hub for Pandemic and Epidemic Surveillance.^8^ The survey was part of a wider exploration of IDS undertaken by IANPHI that is reported elsewhere.^9^

### Survey questionnaire development

A bespoke conceptual model was used for the development of the survey. The conceptual model was informed by WHO’s IDSR,^3^ a well-established framework for the integration of surveillance data, and Morgan et al.’s principles for the development of surveillance systems of the future^5^ building on the lessons to be learned from the international response to the COVID-19 pandemic. The model consists of five key domains: governance, system and structure, financing, core functions, and resourcing requirements (Supplementary File 1, Fig. 1).

Survey questions explored current surveillance systems in IANPHI member countries and the self-reported maturity status of IDS. In countries where IDS systems were implemented, survey questions examined the five domains of the conceptual model. The IDS systems were defined according to the definition of integrated surveillance set out by Nsubuga et al.; ‘a combination of active and passive systems using a single infrastructure that gathers information about multiple diseases or behaviours of interest.’^4^ Respondents were asked to select their IDS maturity status (fully developed, partially developed, or no IDS system).^4^ Respondents without IDS were asked the reasons why IDS had not been implemented and about future plans for its implementation. The survey also collected qualitative data to explore the influence of COVID-19 on IDS development and asked respondents to identify areas of innovative surveillance practice. To support the consistent interpretation of questions, definitions were provided for concepts explored in the survey.

The survey was written in English and translated into Arabic, French, Portuguese, and Spanish. Members of the study team who were native speakers of Arabic, French and Spanish were able to check the translations provided in those languages, and the survey was piloted in all five languages. Before launch, the survey was reviewed by the IANPHI IDS Technical and Executive Committees.^9^ The final survey included 84 structured and 19 open-ended questions (Supplementary File 2).

### Survey completion

The survey was deployed using a web-based survey tool (Select-Survey v5.0^10^) to IANPHI member institutions and partners. IANPHI requested a senior-level focal individual to act as liaison and coordinator to collate the information. Surveys were completed between July and August 2022. A sample size calculation was not undertaken. All IANPHI member institutions were invited to participate, and email reminders were used to maximise the survey response rate.

### Data governance and analysis

Data were securely collected and stored on a cloud-based server housed in the European Union to which only core survey team members had access.

Survey responses that included more than just the respondent’s organisation and contact details were included in the analysis. In the case of multiple answers from the same IANPHI member institute, a single survey version was created by combination where responses were consistent or did not overlap. Where responses conflicted, we retained the answer provided by the more senior respondent in the IANPHI member organisation.

Quantitative descriptive analyses were undertaken using R Statistical Software v3.6.3^11^ and Stata v14.^12^ Depending on the questions, responses were summarised into tables and charts for all respondents or stratified by WHO region,^13^ World Bank Income Group,^14^ and self-reported IDS maturity status. Data were tabulated by responding institution and presented as absolute numbers and proportions. As the denominator used to calculate proportions varied by question, denominators are presented alongside proportions. We highlight differences by stratifiers where substantial differences (approximately 20% absolute percentage points) or clear trends by category were observed. Free text responses were analysed qualitatively to explore the most common views/practices of respondents and emerging issues.

## Results

**Fig. 1.**
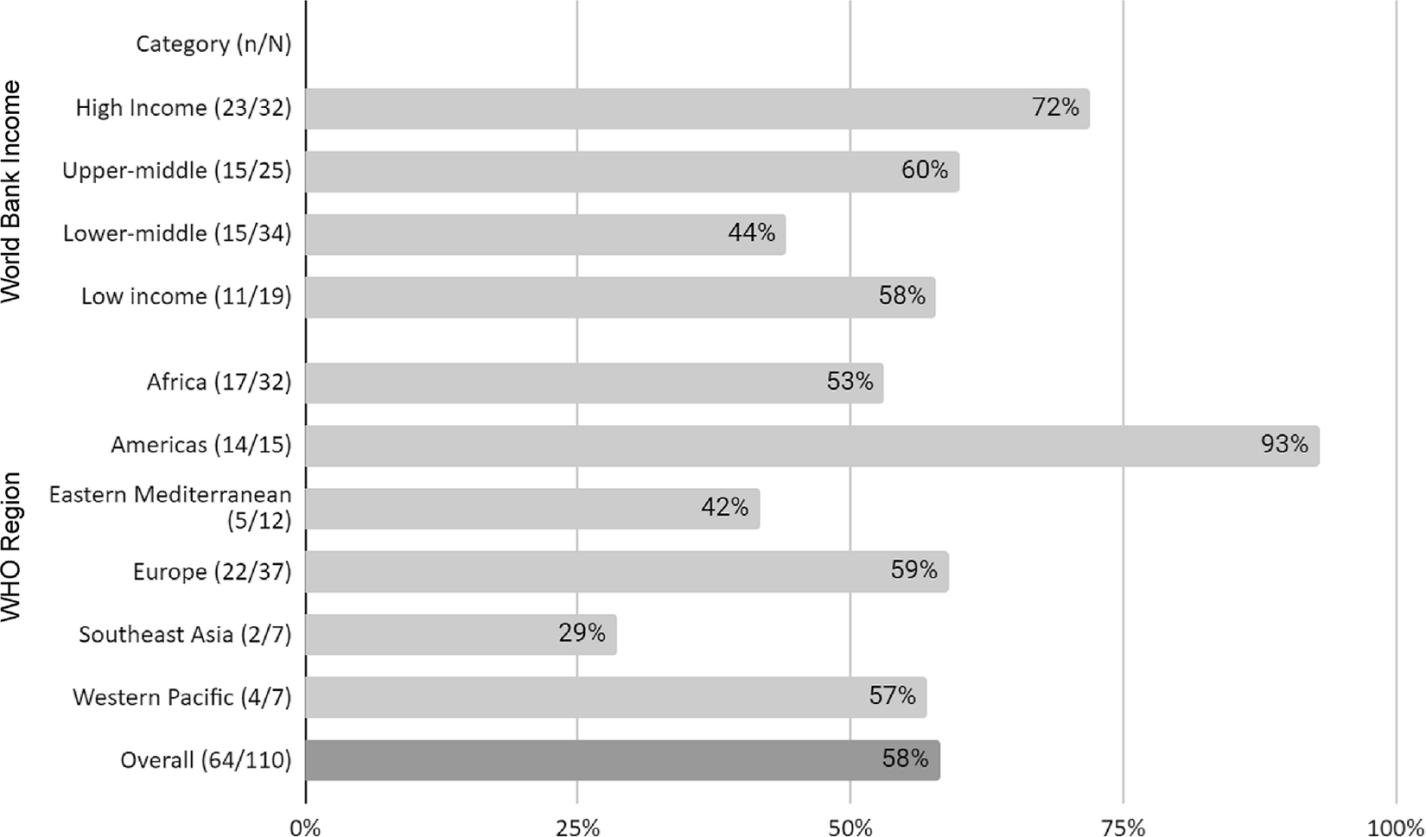
Survey responses as a proportion of IANPHI member institutions* by World Bank income group and WHO region. *Does not include CARPHA, which is an IANPHI regional affiliate.

Of 110 IANPHI member institutions that were sent a survey, 65 (59%) provided a response. As some countries host more than one IANPHI member institute, four countries had responses from two different IANPHI member institutes and the Caribbean Public Health Agency completed the survey representing 24 Caribbean member states. Institutions in the Americas (93%, 14/15) and high-income countries (HICs; 72%, 23/32) were better represented compared with the overall distribution of IANPHI institutions (Fig. 1).

### Current state of surveillance systems

All respondents indicated surveillance systems were in place to monitor notifiable diseases, vaccination coverage, and disease specific and sentinel surveillance systems. Wastewater (78%; 47/60), community-based (77%; 46/60), and behavioural (62%; 35/56) surveillance were described less often (S Table 1). Both public and private healthcare providers, animal health sector, public health sector, and laboratories were frequently described as part of the national surveillance system (S Table 2).

There were differences by level of country income and self-reported IDS maturity status. Compared with HICs, the private sector, pharmaceutical industry, and food safety sector were less likely to contribute to surveillance in low-income countries (LIC) and lower-middle-income countries (LMICs; S Table 2). Conversely, in HICs, community surveillance was less common. Multisectoral surveillance involvement was more often reported in countries with developed or partially developed IDS.

Indicator-based surveillance (IBS) data were most commonly collected using a hybrid of paper and electronic systems (S Table 3). Electronic systems were the most common mechanism for data transfer between agencies (89%, 54/61; S Table 4).

Approximately half of the respondents indicated privacy protection for surveillance as well-established (49%, 31/63), with the remaining half stating privacy protection was either partially developed (41%, 26/63) or not in place for 10% (6/63). Twenty-five percent (3/12) of respondents without IDS reported having no privacy protections in place, and privacy protections were less common in LICs (S Table 5).

Surveillance workforce capacity was most frequently described as average for both event-based surveillance (EBS; 70%, 44/63) and IBS (83%, 50/60; S Table 6). Weak capacity was reported more frequently for EBS (24%, 15/63) than for IBS (8%, 5/60). Workforce capacity gaps were described, including data science and analytics (86%, 56/65) and information technology (IT; 75%, 49/65). Countries without IDS more frequently reported workforce weaknesses for IT and lower-income settings reported greater gaps in their laboratory workforce compared with those with IDS and HICs respectively (S Table 7). Almost all respondents (94%, 61/65) reported surveillance workforce initiatives, either in place or under development (S Table 8).

Ministries of Health (MOH) were most likely to have sole (52%, 33/64) or shared (19%, 12/64) legal mandate to collect data on notifiable diseases (S Table 9). Communicable human (98%, 61/62) and animal diseases (76%, 47/62) were the most frequently mandated notifiable conditions, although approximately half indicated legally mandated reporting of some environmental, chemical, or biological hazards (S Table 10). Only 39% of respondents (23/59) reported good adherence to reporting mandates. Reported adherence was greater in wealthier countries and in countries with some form of IDS system compared with LICs and those without IDS, respectively (S Table 11).

While MOHs most commonly led surveillance either solely or in collaboration with other institutions, one-third of respondents indicated that the NPHI was solely responsible. NPHI leadership was more frequently described in HICs, while the MOH led more often in LICs, particularly in Africa and the Americas (S Table 12). NPHIs had sole (17%, 11/64) or joint (19%, 12/64) legal mandates for notifiable disease or hazard reporting in their country (S Table 9).

Where IDS was in place, the NPHI had sole (20%, 10/50) or joint (60%, 30/50) responsibility for IDS in most countries, typically sharing responsibility with the MOH (S Table 13). Core functions of IDS (including case/event detection, reporting, investigation, analysis, response, feedback, evaluation, and preparedness) were typically led by NPHIs, with the MOH more frequently sharing leadership with the NPHI on preparedness and response (S Table 14). Workforce development initiatives were predominantly led by the MOH (60%; 39/65) or NPHI (58%; 38/65; S Table 8).

### Integrated disease surveillance

**Fig. 2.**
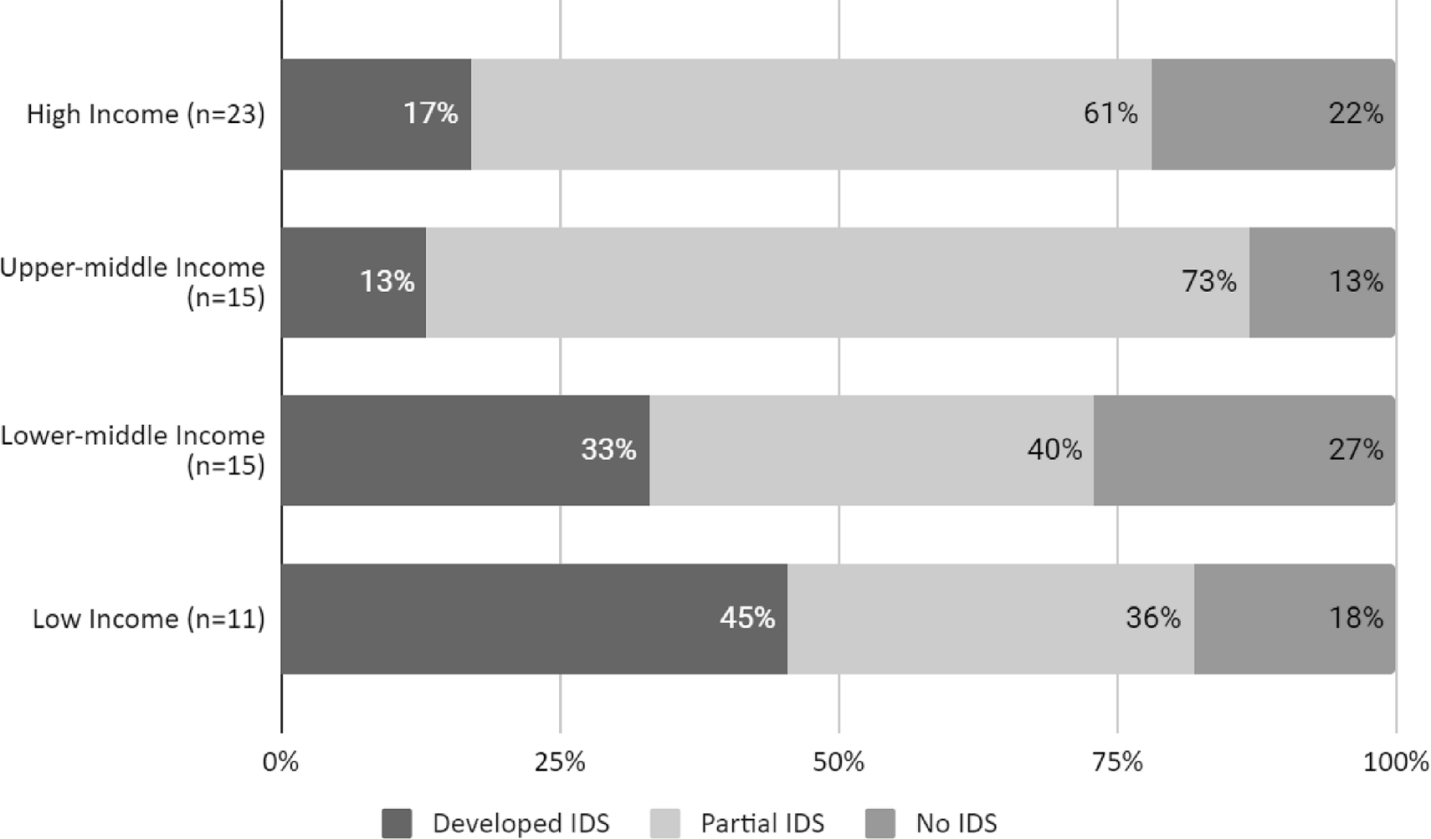
Relationship between reported IDS maturity and country income group.

Using Nsubuga et al.’s definition, most respondents report a partially developed IDS (55%; *n* = 36) vs a fully developed one (25%; *n* = 16) or no IDS in place (20%; *n* = 13). LICs were more likely to report developed IDS than wealthier countries (Fig. 2, S Table 15). All 10 of the LICs and LMICs with a developed IDS were in the African region.

**Fig. 3.**
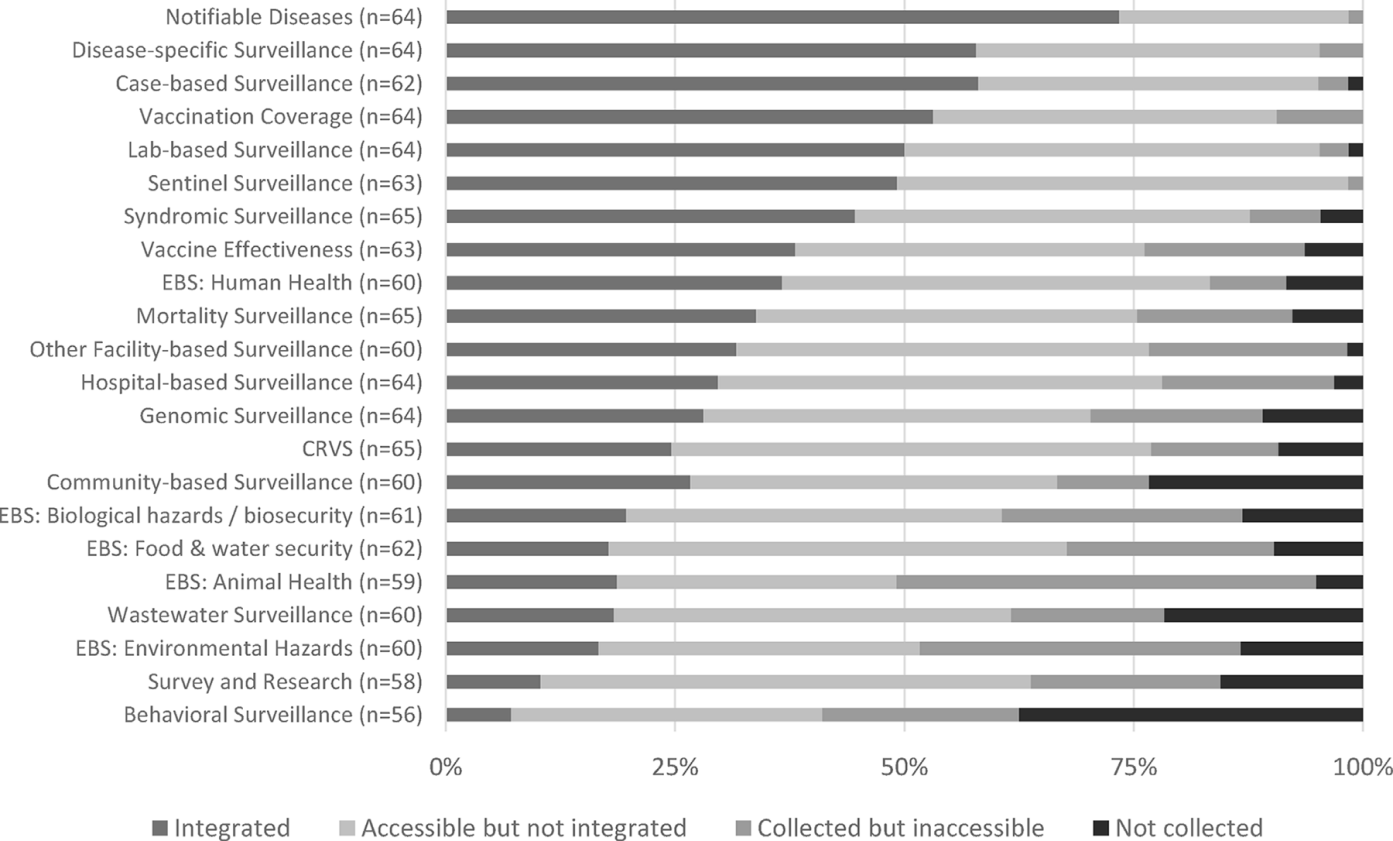
Level of data integration (as a proportion of responses) of surveillance systems. In a limited number of cases, respondents indicated data were collected but insufficient information was provided to determine if data were accessible and/or integrated. In these events, the case has been classified as ‘collected but inaccessible’ as the most conservative degree of data integration based on response.

Surveillance data from specific systems, notably environmental, animal health, behavioural surveillance, and surveys were often reported to be inaccessible to the lead public health agency, even when institutions reported partial or fully developed IDS systems. Examining specific data collection systems, fewer than half of respondents reported data were integrated into their national public health surveillance system. The exceptions were notifiable, case based, disease specific, and vaccination coverage surveillance systems where a majority of respondents indicated these systems were integrated (Fig. 3, S Tables 16–18).

Poor sectoral integration was more commonly reported in countries with partial IDS (compared with a developed IDS system), with more than half reporting that it was not possible to integrate data from outside human healthcare sectors (e.g. animal health, environmental health, and agricultural sectors) due to lack of interoperability, data sharing agreements, or other barriers (S Table 19). Half of the respondents with a developed (6/16) or partial (20/35) IDS reported that data on non-communicable diseases could not be integrated (S Table 20). Conversely, approximately half of respondents in countries without IDS indicated using data from multiple sources for public health action (S Table 21).

Most respondents with full or partial IDS reported at least a moderately strong ability to perform the following surveillance core functions: detection and reporting of events/indicators (*detect*), investigation and/or verification of events (*investigate*), data analysis and reporting (*analyse*), and public health event response (*respond*; Table 1). Weaker performance was reported for the core function: evaluation and provision of feedback for system improvement. From a system perspective, greater variability in the ability to perform core functions was observed in LICs, with more respondents indicating weaker systems compared with HICs (S Fig. 2).

Laboratory integration is key to the function of IDS. Almost all respondents with IDS had access to national public health laboratories (NPHL) data (98%, 51/52; S Table 22). Respondents in wealthier countries were more likely to report the integration of private laboratory data (HICs: 78%, 14/18; Upper middle income countries (UMICs): 69%, 9/13; S Table 22) and data transfer through compatible IT systems (HICs: 88%, 15/17; UMICs 77%, 10/13), whereas laboratory data in LICs were less likely to be integrated (LICs: 22%, 2/9; LMIC: 36%, 4/11; S Table 23). In countries with IDS, 88% (43/49) reported genomic testing/sequencing available, primarily through NPHLs (S Table 24).

Many respondents stated that COVID-19 response activities had strengthened national surveillance by advancing laboratory and genomic surveillance, promoting the development or expansion of electronic structures, optimising systems for data sharing or integration, and facilitating multisectoral engagement. Further benefits included catalysing the development of new systems, including case-based reporting, vaccination, and border health surveillance systems.

### Barriers to integrated disease surveillance

**Fig. 4.**
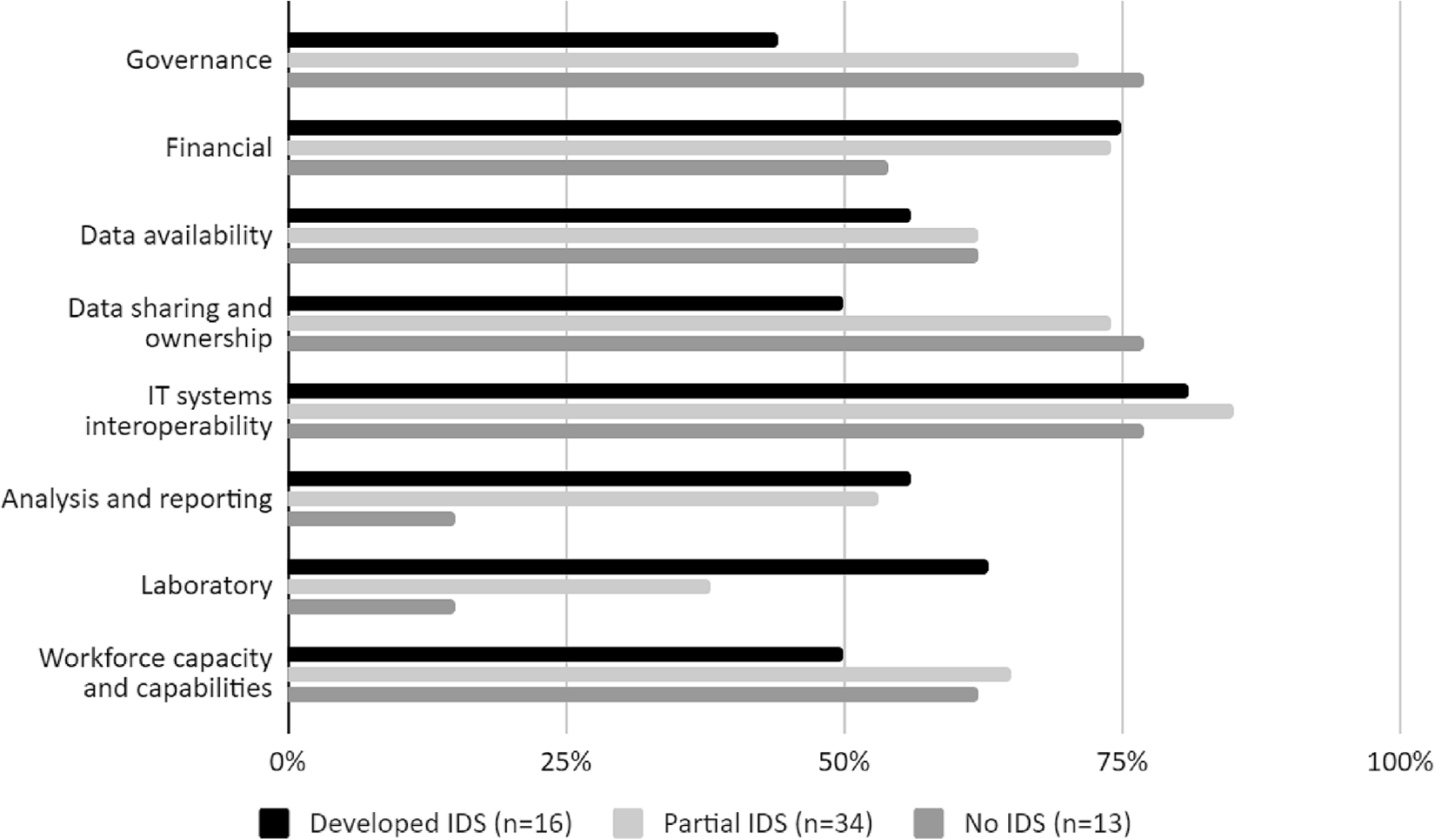
Barriers to integrated disease surveillance. In countries with either a developed or partially developed IDS system, these factors reflect challenges experienced in setting up and running the IDS system. In countries without an IDS system, these are factors that have prevented the establishment of an IDS system. Factors included governance (leadership, accountability, regulation, and enforcement); finance (inadequate investment, multiyear budget not available); data availability (requisite data not collected, not collected to a suitably high standard, or not shared by the organisations who are responsible for collecting that data); data sharing and ownership (lack of involvement, unclear roles and responsibilities, internal politics, unclear lines of reporting and accountability, territorialism, conflict/uncertainty re: intended use of data); IT systems (incompatible IT systems to migrate data, migration from paper to electronic format, suboptimal IT systems, data security, data protection); analysis and reporting (lack of statistical package, unavailability of big data analysis); laboratory (lack of testing capabilities, lack of multisectoral reporting, lack of provider reporting); and workforce capacity and capabilities (lack of multisectoral training, lack of analytical skills, lack of data collection skills).

The most frequently reported challenges to establishing IDS included IT system issues (84%, 42/50), financial constraints (74%, 37/50), data sharing and ownership limitations (66%, 33/50), workforce capacity gaps (60%, 30/50) and data availability (60%, 30/50; Fig. 4, S Table 25). In countries without IDS, similar barriers existed and included IT systems (77%, 10/13), data sharing and ownership (77%, 10/13), and governance (77%,10/13; S Table 26). In countries without IDS, priorities included the integration of surveillance data at both national and subnational levels and the development of requisite surveillance IT and digital infrastructure (S Table 27).

Lack of sustained, multiyear financing was found to be a constraint across regions. Based on the survey results, government financing was identified as the most common source of IDS funding (77%, 37/48; S Table 28). In countries without IDS, 30% (3/10) of respondents were exploring international non-governmental organisation funding for IDS (data not shown).

Additional barriers to surveillance data integration noted in the open-ended responses included parallel systems, sectoral siloes, lack of coordination, decentralised systems, and legal limitations on the collection, sharing, and integration of personally identifiable data. Clearer mandates, better defined roles and coordination, workforce strengthening, and the development of a policy framework and interoperable information systems were proposed as mechanisms to address the gaps.

Even in countries with IDS, data from some sectors, agencies, or sources cannot be integrated due to a lack of interoperability or data sharing. Regardless of the level of IDS development, most respondents reported some disparities in semantic consistency (i.e. the delivery and interpretation of data in a standard way across systems;^15^ S Table 29). Laboratory-specific barriers to integration included poor data systems/integration (54%, 27/50), followed by lack of equipment/supplies (40%, 20/50), limited staff (40%, 20/50), and inefficient specimen transfer (18%, 9/50; S Table 30).

## Discussion

Our survey data suggest that public health surveillance systems across the globe, although built on similar principles, are at different levels of maturity but face similar developmental challenges. Our findings corroborate the key principles proposed by Morgan et al.,^5^ which suggest that the following are necessary components for effective surveillance: leadership, ownership and governance, supporting legal mandates and regulations, adherence to mandates, and enforcement of regulations.

We found that the implementation of IDS systems differed across country settings, and survey responses provided useful insights into the process of surveillance system integration. Integration can be seen from a ‘whole systems perspective’ that extends beyond just the technical integration of databases. Our findings suggest that the integration of surveillance systems is complex, involving multiple stakeholders and sectors, necessitating action at all levels of the health system. From the surveillance system perspective, integration as a driver of more timely decision-making and better public health outcomes was a key consideration, for which the system needs to be agile, responsive, and resilient.

Possibly driven by differences in the way IDS is understood, the self-reported level of IDS maturity did not necessarily mirror the level of country income. LICs were more likely to report developed IDS, but they tended to be African countries with greater familiarity with the IDSR strategy^3^ promoted by the WHO across the African region for over two decades.^16^ We also found conflicting responses from respondents who self-classified their IDS as ‘fully developed’ but also reported having gaps in specific sectors and surveillance mechanisms.

Our study found that whilst most systems were overseen by the MOH, in many countries, NPHIs played a significant role in integrated disease surveillance, leading on core surveillance functions, including the detection and investigation of public health events and analysis of public health data. This was especially true for HICs and countries with more developed IDS. As has been noted elsewhere in the context of COVID-19 surveillance,^17^ this suggests an important role for NPHIs in the development and functioning of IDS. It may also reflect, both historically and in relation to COVID-19,^18–20^ that the creation of an NPHI in a country is part of an enhanced strategy towards better disease control, prevention, and response and therefore an indicator of a more mature public health system.

Regardless of the country-income level, there was consensus that sustainable, sufficient, and longer-term resourcing are critical for effective surveillance. Consistent with the calls Morgan et al. make for adequate financing,^5^ responses identify the importance of resourcing for laboratory, genomic, and IT infrastructure but also a skilled workforce necessary to support these different parts of the surveillance system. As seen during COVID-19,^21^ technology and the supporting workforce enable better disease surveillance integration through greater automation of data collection, management, analysis, and information and knowledge transfer. Our findings indicate that skilled surveillance workforces are not evenly distributed geographically, across different surveillance systems, and sectors. The initiatives set out in the WHO’s public health and emergency workforce roadmap present an opportunity for countries looking to strengthen their surveillance workforce through collaboration, shared learning, and peer support.^22^

Despite acknowledgement of the need for sustainable, longterm resourcing, many respondents to our survey reported short-term and insufficient financing of IDS. This was the case for many respondents from LMICs and LICs who are often highly dependent on international aid and vertical programmes. LIC and LMIC respondents suggested that whilst they may have the capacity to maintain IBS, they may not have the capabilities and resources to invest in or maintain EBS. Previous research has indicated that external donor funding has not been found to be sustainable and LIC and LMIC respondents favour siloed vertical surveillance that is often incompatible with efforts to integrate across systems.^23,24^

Perhaps unsurprisingly, given that they are recognised building blocks of strong and effective health systems,^25^ our findings indicated that limitations related to governance, adherence to legal mandates, and infrastructural barriers were all perceived as barriers to effective surveillance integration. Countries with more developed IDS tended to report fewer challenges related to governance.

Consistent with a review of the integration between human and animal health surveillance,^15^ our survey identified the importance of semantic consistency and functioning at the interfaces between surveillance systems. There were challenges integrating data from NGOs, private, academic, and pharmaceutical sectors, especially in LICs and LMICs. As noted elsewhere,^15,26^ challenges at the interface between surveillance systems were more common for non-human health sectors (e.g. environmental health and animal health sectors) and non-infectious disease sectors (e.g. non-communicable diseases and occupational health). NPHIs are likely to be well positioned to work collaboratively across sectors to address these challenges. Global strategies designed to promote the integration of surveillance data across human and non-human health sectors, such as the One Health Quadripartite’s ‘Joint Plan of Action’,^27^ will also require support from NPHIs in order to succeed.

Data protection was a common issue. As data transparency and the social acceptability of data collection and use is dependent upon privacy protection, attention will be needed both to enhance public trust in IDS and the public institutions which have responsibility for it.

### Strengths and limitations

Our study provides insight into the state of surveillance systems and IDS at a time, in the aftermath of COVID-19, when the international community is re-evaluating functions and systems related to epidemic and pandemic preparedness and response. The study leveraged IANPHI’s unique ability to pull together the collective experience and insights of its constituent NPHIs globally.^7^ All the respondents were senior-level representatives who were well placed within their respective public health systems to provide a country-level overview. The survey achieved a good response rate not commonly attained in multicountry surveys with representation across all WHO regions and World Bank income groups.

All self-administered surveys are limited by the perceptions and experiences of respondents to interpret the questions and response options. This was addressed this through a pilot process aimed to increase clarity, and provided the survey in multiple languages, and with examples and definitions to help convey meaning. However, not all respondents could complete the study in their own language and some concepts (e.g. ‘IDS status, IDS maturity’) were self-determined and may have introduced interpretation biases.

We have not explored in detail the challenges associated with interoperability and data sharing which we report. As this is a critical dimension of integrated and collaborative surveillance, further research should in particular focus on this specific dimension.

This study was also prone to sampling bias, inherent from surveying only members from IANPHI. As such, the views of countries without an NPHI are underrepresented. Despite targeting senior-level respondents, having only one focal person per NPHI may have limited knowledge on specific topics, other organisations, or sectors. As national representatives, respondents may be less familiar with local, district, or provincial contexts. Some WHO regions were also underrepresented (e.g. Western Pacific, Eastern Mediterranean, and Southeast Asia).

### Implications for practice

Defining more clearly the purpose of IDS, its functions, and mandate-based on intended public health outcomes will likely be an important aspect of international IDS efforts and collaborations to strengthen global health security. Improving multisectoral policy decisions and responses for early warning, preparedness, response, and recovery from epidemics and pandemics can offer better and more integrated health improvements. Improving governance and legal enablers through infrastructures, processes, and resourcing may be beneficial for achieving integration objectives. Governments may find it beneficial to consider sufficiently resourcing surveillance systems and investing in a sustainable way in order to ensure robust IDS systems that can perform across sectors. Sustainable workforce development (both capacity and skills development) as well as for laboratory, genomics, and IT interoperable infrastructures are essential components of IDS systems. Tackling weaknesses in monitoring and evaluation and feedback have been identified as essential for quality improvements and assurance and the creation of learning systems.

Our findings corroborate knowledge gaps across issues related to surveillance, ranging from optimal configuration, processes to the effectiveness of its various components, as reported elsewhere.^6^ Further research would be beneficial for building the evidence base to improve the effectiveness and efficiency of IDS for better response. This includes research on the role of NPHIs, exploration of multisectoral barriers and opportunities, the value of integration of certain sectors (e.g. One Health and non-health sectors), and the understanding of the influence and impact of different country contexts and challenges at the implementation level. Wider communities of practice are therefore needed to bridge these interface divides. NPHIs, as system enablers, appear to offer value in their leadership capacity and ability to use their convening powers to establish and maintain such networks. The development of an NPHI in each country may be considered as a complementary investment for supporting and enabling stronger disease surveillance systems.

## Supplementary Material

Supplementary Files

## Figures and Tables

**Table 1:** Self-assessment of core surveillance function performance by IDS status.

a) Developed IDS systems						

Core functions	Weak	Moderate-weak	Moderate	Moderate-strong	Strong	*n*

Detect	6%	13%	13%	44%	25%	16
Investigate	0%	6%	38%	25%	31%	16
Analyse	0%	6%	25%	38%	31%	16
Respond	0%	0%	6%	56%	38%	16
Evaluate and provide feedback	0%	20%	33%	20%	27%	15

b) Partial IDS systems						

Core functions	Weak	Moderate-weak	Moderate	Moderate-strong	Strong	*n*

Detect	9%	18%	18%	44%	12%	34
Investigate	9%	14%	20%	37%	20%	35
Analyse	11%	9%	17%	43%	20%	35
Respond	14%	6%	11%	51%	17%	35
Evaluate and provide feedback	27%	24%	33%	6%	9%	34

Definition of functions and classification of performance:	

**Detect: ability to detect and report events/indicators**	
Weak	1) National strategy, guidelines, and/or Standard Operating Procedures (SOPs) for surveillance are not available or under development
Moderate-weak	2) National strategy, guidelines, and/or SOPs for surveillance have been developed but not implemented. The surveillance system is functioning but lacks systematic immediate reporting or weekly reporting of events and/or data
Moderate	3) National strategy, guidelines, and/or SOPs for surveillance have been developed and are being implemented at the national level. The surveillance system provides immediate and weekly reporting of events and/or data with lab results integrated.
Moderate-strong	4) National strategy, guidelines, and/or SOPs for surveillance have been developed and are being implemented at the national and intermediate levels. The surveillance system provides immediate and weekly reporting of events and/or data with laboratory results integrated and integration between IBS and EBS
Strong	5) National strategy, guidelines, and/or SOPs for surveillance linking all sectors have been developed and implemented at national, intermediate, and primary public health levels; and the system is exercised (as applicable), reviewed, evaluated, and updated on a regular basis, with improvement at all levels in the country, with all components linked to one national surveillance system
**Investigate: ability to investigate and/or verify events**	
Weak	1) Method, process, or mechanisms for verifying and investigating detected events is not available or under development
Moderate-weak	2) Method, process, or mechanisms for verifying and investigating detected events has been developed but not implemented
Moderate	3) Method, process, or mechanisms for verifying and investigating detected events has been developed and is being implemented at the national and intermediate levels
Moderate-strong	4) Method, process, or mechanisms for verifying, investigating and risk assessing detected events has been developed and is being implemented at the national and intermediate levels, involving trained personnel from multiple sectors
Strong	5) Method, process or mechanisms for verifying, investigating, and risk assessing detected events is being implemented at national, intermediate, and primary public health levels, involving trained personnel from multiple sectors and exercised (as applicable), reviewed, evaluated, and updated on a regular basis
**Analyse: Ability to analyse data and report results**	
Weak	1) Surveillance data are received sporadically and analysed on some priority diseases, or unusual events, often with a delay
Moderate-weak	2) Surveillance data are received regularly (i.e. weekly and/or monthly). An ad hoc team does some analysis of data
Moderate	3) Surveillance data are received regularly and analysed on some priority diseases, or unusual events, often with delay. Data are shared across sectors.
Moderate-strong	4) Surveillance data are received and analysed regularly. Epidemiological bulletins are generated and disseminated across sectors and internationally on a regular basis. Data are shared across sectors and internationally on a regular basis
Strong	5) Surveillance data analysis is conducted, and epidemiological bulletins are generated and disseminated across sectors and internationally on a regular basis. An electronic platform and a dedicated team support data management and the generation of epidemiological bulletins. Data are shared across sectors and internationally on a regular basis. Capacity for advanced data analysis is ensured
**Respond: ability to respond to a public health event**	
Weak	1) National strategy, guidelines, and/or SOPs for IDS in response to a public health event are not available or under development
Moderate-weak	2) National strategy, guidelines, and/or SOPs for IDS in response to a public health event have been developed but not implemented
Moderate	3) IDS system produces standard data for use in response at a national level
Moderate-strong	4) IDS system has the capacity to adapt or expand data collection and reporting in response to a public health event. Data for response are available at a national and intermediate levels.
Strong	5) IDS system has mechanisms in place to quickly adapt or expand data collection and reporting in response to a public health event. Surveillance data are routinely used in forecasting. Data for response are available at a national, intermediate, and primary public health levels.
**Evaluate: ability to evaluate and provide feedback for system improvement**	
Weak	1) National strategy, guidelines and/or SOPs for IDS system monitoring and evaluation are not available or under development
Moderate-weak	2) National strategy, guidelines and/or SOPs for IDS system monitoring and evaluation have been developed but not implemented
Moderate	3) IDS system evaluation conducted and/or monitoring data reviewed at a national level at least once a year
Moderate-strong	4) IDS system evaluation conducted and/or monitoring data reviewed at a national level and intermediate level multiple times per year
Strong	5) IDS system monitoring and evaluation conducted continuously at a national, intermediate, and primary public health level and routinely applied for system strengthening
