# Supplementary material for: Surveillance system integration: reporting the results of a global multicountry survey
Source: Public Health. Author manuscript; Available in PMC 2024 Aug 1. (PMC11292781; doi:10.1016/j.puhe.2024.03.004)
Supplement: Supplementary Files [file NIHMS2009509-supplement-Supplementary_Files.zip › 1-s2.0-S0033350624001069-mmc2.pdf]

IANPHI Integrated Disease Surveillance Country Survey - English

Page 1

Public Health Institutes of the World
IANPHI

Welcome to the International Association of National Public Health Institutes (IANPHI) Integrated Disease Surveillance (IDS) Country Survey.

The survey will ask you, as the representative for your country, to answer a series of questions related to the surveillance systems in place in your country, at national and sub-national levels, and the extent to which they are integrated.

The survey should take between 30 and 40 minutes to complete. You are able to complete the survey in more than one session by using the 'log out' button on each page. To log back in to the survey, use the code: **IANPHI2022**

If you have any questions about this survey, please don't hesitate to contact us on
IDS.Survey@ianphi.org

This survey is available in five languages: English, French, Spanish, Portuguese, and Arabic. These can be found at the following links:

English: http://43.selectsurvey.net/ianphi/TakeSurvey.aspx?SurveyID=IANPHI2022
Arabic: http://43.selectsurvey.net/ianphi/TakeSurvey.aspx?SurveyID=7lKH859
French: http://43.selectsurvey.net/ianphi/TakeSurvey.aspx?SurveyID=7lKH959
Spanish: http://43.selectsurvey.net/ianphi/TakeSurvey.aspx?SurveyID=7lKHl59
Portuguese: http://43.selectsurvey.net/ianphi/TakeSurvey.aspx?SurveyID=7lKHm59

Introduction to IANPHI's Integrated Disease Surveillance Global Survey

Thank you for agreeing to complete this survey as the on behalf of your country, as the nominated IANPHI focal point.

IANPHI has been awarded a grant by the Bill and Melinda Gates Foundation to provide an understanding of country level surveillance and integration diseases surveillance (IDS) systems. This project will also support the WHO Hub for Pandemic and Epidemic Intelligence in Berlin in the next stage of implementation of their strategy 'Pandemic and Epidemic surveillance; better data, better analytics, better decisions'.

The focus of this survey is on the surveillance and the integration of surveillance data and systems. The questions seek to understand the extent to which the agency which oversees public health surveillance in your country is able to access and integrate data in the detection, response and management of public health threats.

By completing this survey you are consenting for the data to be analysed and used by IANPHI. The data and findings from this survey will be used for the following purposes:

1. A report to the Gates Foundation on evidence and outcomes with recommendations to support National Public Health Institutes (NPHIs) to develop robust, resilient, and sustainable integrated disease surveillance, reporting and response systems.
2. To support the development of a roadmap for the WHO Hub-Berlin on learnings, evidence and recommendations for priority setting. This will include outlining essential areas of fundamental development and focus areas for NPHIs in different stages of IDS system development.
3. To provide evidence to support the development of IANPHI guidelines for NPHIs reviewing, planning or developing IDS.
4. To generate publications by IANPHI as the association representing IANPHI members

The survey has been determined to be exempt from requiring Institutional Ethics Review Board approval by Emory Universities Institutional Ethics Review Board. Further details are available upon request.

Responding to the Integrated Disease Surveillance Global Survey

As the nominated IANPHI focal point, **you are the only person in your country who has been asked to respond to this survey. We ask that you complete the survey for your country as a whole, incorporating information relevant to every level of surveillance system in operation.**

As the questions within this survey are designed to collect a information related to surveillance and IDS systems across the country, they may require you to consult with colleagues and those working in organisations outside of your own.

The survey allows you to complete the responses across several sessions in order to facilitate information gathering and liaison with colleagues. You can logout of the survey at any stage. Your responses will be saved and you will be able to log back in using the code:
IANPHI2022

The survey should take between 30 and 40 minutes to complete.

We would be grateful if you could complete the survey before Monday 8th August 2022.

Once the survey is closed and the analysis is complete, IANPHI will share the final report with all IANPHI members.

If you have any questions related to the survey, please do not hesitate to get in contact with us using the following email address: IDS.Survey@ianphi.org

Highlighting innovative practice

We also wish to highlight areas where innovative approaches or practice to integration of data and analysis have enabled collection, sharing, reporting and rapid prediction and decisions. **If have case studies or examples you would like to share with us and other IANPHI members, please ensure you complete the 'Integration of surveillance systems: examples of innovative practice' section on the penultimate page of the survey.**

Whilst the data collected as part of this survey will be anonymised, we may wish to highlight the areas of innovative practice which you share with us in our reports, publications or elsewhere. If this is the case, and we wish to use country specific information you provide to highlight innovative practice, we will contact you for permission beforehand.

1. Please tick here to indicate that you understand the information provided above, and consent to taking part in the survey as the representative for your country.

   *

   ☐ I consent to taking part in the IANPHI IDS Country Survey

Public Health Institutes of the World
IANPHI

Survey Respondent Details

2. What is your name?

3. What is your job title or role?

4. What is your email address?*

5. What is your telephone number (including international dialing code)?

6. What is the name of the organisation that you work for?*

7. What is the address of the organisation that you work for?

8. What type of organisation do you work for?*
- ○ National Public Health Institute
- ○ Ministry of Health
- ○ National Public Health Laboratory (not affiliated to the National Public Health Institute of Health)
- ○ Other Government Department
- ○ Other organisation, please specify

Public Health Institutes of the World
IANPHI

Surveillance Systems

The following questions ask about surveillance systems in your country, the types of surveillance systems that are in place, the sectors that are involved, the type of data that is collected, and the legal and workforce structure that is in place to support them.

The questions below also explore the integration of surveillance systems.

One definition of what constitutes a fully integrated surveillance system is provided by Morgan et al.(2021), who describe the integration of surveillance systems as follows.

"Multiple surveillance systems can be integrated on...a population-[based] representative foundation, according to the priorities of the country and leveraging internal resources, such as surveillance programmes run by academic and non-governmental institutions.

A fully integrated surveillance system could include integrated disease surveillance and response, including ... case reporting; pathology-based cause of death surveillance; electronic health and laboratory record data transfer; serological surveillance; vaccine adverse events reporting; epizootic and food safety surveillance systems on the One Health model; participatory community surveillance; and disease-specific systems for HIV, tuberculosis, malaria, vaccine-preventable diseases, [data linkage] and many others."

Whilst this definition describes a fully integrated surveillance system, **countries are likely to have surveillance systems at different stages of development and integration.** This is one of the things we are keen to explore in this survey.

The overarching aim of these questions is to understand the extent to which the agency which oversees the surveillance systems in country has access to the data collected by those systems, and integrates that data, and / or the sharing of information from analysis of that data, in the detection, response and management of public health threats.

9. Which is the **lead agency** responsible for public health surveillance system in your country?
   - ○ National Public Health Institute (NPHI)
   - ○ Ministry of Health (MoH)
   - ○ Joint ownership - NPHI and MoH
   - ○ Centre for statistics
   - ○ Other, please specify

10. Which of the following surveillance systems in your country does the lead agency responsible for surveillance have access to? How well integrated is the data from each surveillance system in to the detection and response to threats to public health?

    Your answer should correspond to one of the following options:

    1. **Lead agency has access, data integrated:** this answer indicates that the lead agency for the surveillance system in your country (NPHI, MoH etc.) has access to this surveillance data and integrates it with other surveillance systems and data sources to support the detection and management of public health threats
    2. **Lead agency has access, data not integrated:** this answer indicates that the lead agency for the surveillance system in your country (NPHI, MoH etc.) has access to this surveillance data but does not integrate it with other surveillance systems and data sources support the detection and management of public health threats.
    3. **Lead agency has no access to data:** this answer indicates that the lead agency for the surveillance systemin your country (NPHI, MoH etc.) does not have access to this surveillance data
    4. **Data not collected:** this answer indicates that this data is not collected in your country
    5. **Unknown:** this answer indicates that you do not have the information needed to answer the question related to this surveillance system
    6. **Other:** where none of the other options are applicable, please indicate and provide further details in the comments box below.

Select all that apply.

	Lead agency has access, data integrated	Lead agency has access, data not integrated	Lead agency has no access to data	Data not collected	Unknown	Other
Notifiable disease surveillance systems	○	○	○	○	○	○
Syndromic surveillance	○	○	○	○	○	○
Sentinel surveillance	○	○	○	○	○	○
Laboratory based surveillance	○	○	○	○	○	○
Hospital-based surveillance (e.g. admissions, discharges, emergency unit)	○	○	○	○	○	○
Other health facility based surveillance (e.g. primiary care, clinics)	○	○	○	○	○	○
Case-based surveillance (information about a case or person diagnosed with a disease or condition that poses a threat to health)	○	○	○	○	○	○
Disease-specific surveillance (e.g. TB, HIV/AIDS)	○	○	○	○	○	○
Community-based surveillance	○	○	○	○	○	○
Mortality surveillance	○	○	○	○	○	○
Genomic surveillance	○	○	○	○	○	○
Behavioural surveillance	○	○	○	○	○	○
Vaccine coverage	○	○	○	○	○	○
Vaccine effectiveness /side effects	○	○	○	○	○	○
Surveys and research of public opinion	○	○	○	○	○	○
Health and demographic surveillance system (e.g. using civil registration and vital statistics (CRVS))	○	○	○	○	○	○
Waste water surveillance	○	○	○	○	○	○

Event based surveillance monitoring signals related to human health	○	○	○	○	○	○
Event based surveillance monitoring signals related to the animal health sector (Veterinary and animal health services)	○	○	○	○	○	○
Event based surveillance monitoring signals related to environmental hazards	○	○	○	○	○	○
Event based surveillance monitoring signals related to biological hazards and biosecurity	○	○	○	○	○	○
Event based surveillance monitoring signals related to food and water security	○	○	○	○	○	○
Other (please specify)	○	○	○	○	○	○

11. If other, please provide details.

12. Which sectors are involved in your country's surveillance systems (event-based, indicator-based or other)?

- ☐ Public healthcare providers
- ☐ Private healthcare providers
- ☐ Public health
- ☐ Animal health
- ☐ Agriculture
- ☐ Environmental
- ☐ Laboratory
- ☐ Private sector
- ☐ Communities
- ☐ Pharmaceutical sector
- ☐ Occupational health
- ☐ Chemical and poison sector
- ☐ Disaster management
- ☐ Food safety
- ☐ Biosafety and biosecurity
- ☐ Other, please specify

Integrating surveillance data in the response and management of outbreaks, diseases and other risks to human health

13. Does the current surveillance system in your country allow for the

integration of surveillance data from differentsources in to the **response and management** of outbreaks, diseases and other risks to human health?

- ○ Yes
- ○ No
- ○ Other, please specify

14. Where do you think the gaps, weaknesses and areas for development lie, in your country's ability to integrate surveillance data from different sources in the **response and management** of outbreaks, diseases and other risks to human health?

15. What would be needed to respond to and address the gaps, weaknesses and development opportunities in yourcountry's ability to integrate surveillance data from different sources in the response and management ofoutbreaks, diseases and other risks to human health, as you have outlined above?

Data collection and sharing

16. How is data collected for the indicator-based surveillance programmes you have in your country?

	**Data collected digitally**	**Data collected on paper**	**Data collected digitally and on paper**	**Surveillance system not in place**	**Other, please specify below**
Notifiable disease surveillance	○	○	○	○	○
Mandatory notification	○	○	○	○	○
Sentinel surveillance	○	○	○	○	○
Syndromic surveillance	○	○	○	○	○
Registers (Routine health indicators)	○	○	○	○	○
Mortality data	○	○	○	○	○
Laboratory data	○	○	○	○	○
Surveys and research	○	○	○	○	○
Hospital discharge data	○	○	○	○	○
Genomic surveillance	○	○	○	○	○
Behavioural surveillance	○	○	○	○	○
Vaccine coverage	○	○	○	○	○

17. If other, please provide details below.

18. How is surveillance data transferred between agencies in country?

- ☐ Electronically through compatible IT systems
- ☐ Electronically other (please specify)
- ☐ Telephone-based (text message or fax)

☐ Manually
☐ Data not shared across agencies
☐ Other (please specify below)

19. Please briefly describe how you share surveillance data across agencies in your country.

Legal mandate for surveillance

20. Is there a legal mandate for organisations in your country to report notifiable diseases, hazards or other threats human health?
☐ Yes - to the Ministry of Health
☐ Yes - to the National Public Health Institute
☐ Yes - other, please specify
☐ No
☐ Other, please specify

21. Which diseases, hazards or other threats human health does the legal mandate in your country state must be notified?

Please select answers which apply to one or more diseases, hazards or other threats human health.
☐ Communicable diseases
☐ Non-communicable diseases
☐ Causative organisms
☐ Chemical and radiation hazards
☐ Environmental hazards
☐ Diseases in animals
☐ Other hazards to human health, please specify

22. If the full list of legally mandated notifiable diseases, hazards or other threats to human health is available pleaselist, or share a web link for further information, in the text box below.

23. Does the legal mandate to report for the above notifiable diseases, hazards or other threats human health apply to the following sectors?

	Yes	No	Unknown
Public sector	○	○	○
Private healthcare (e.g. private hospitals, private laboratories)	○	○	○
NGOs (e.g. those delivering heath care services)	○	○	○
Animal and livestock sector	○	○	○
Pharmaceuticals	○	○	○
Agriculture	○	○	○
Food industry	○	○	○
Water	○	○	○
Chemicals and poisons	○	○	○
Occupational health (private sector)	○	○	○

24. How well is the legal mandate for the notification of conditions of hazards adhered to and enforced in your country?

○ Not well adhered to

○ Partially adhered to
○ Well adhered to
○ Uncertain / unknown

25. Is the protection of privacy and and the oversight and review of privacy protection (e.g. by privacy watchdogs) an established part of the public health surveillance systems in your country?

○ Yes – privacy protection and review of surveillance systems is very well established
○ Yes – privacy protection and review of surveillance systems is partially or in the process of being established
○ No – privacy protection and review is not incorporated in to the surveillance systems in my country
○ Other, please specify

Surveillance workforce

26. How would you rate the workforce capacity to support the **indicator based** surveillance systems in your country?

○ Weak - in need of development, few of the requisite skills, competencies and personnel in place
○ Average - some development needed, some of the of the requisite skills, competencies and personnel in place
○ Strong - no further development needed, requisite skills, competencies and personnel in place
○ No indicator based surveillance system in place
○ Other, please specify

27. How would you rate the workforce capacity to support the **event based** surveillance systems in your country?

○ Weak - in need of development, few of the requisite skills, competencies and personnel in place
○ Average - some development needed, some of the of the requisite skills, competencies and personnel in place
○ Strong - no further development needed, requisite skills, competencies and personnel in place
○ No indicator based surveillance system in place
○ Other, please specify

28. Where are the gaps within the capabilities of your surveillance workforce (if any exist)?

☐ Laboratory
☐ Epidemiology
☐ Administrative / data entry
☐ Data science & analytics
☐ Public health generalist
☐ IT
☐ No gaps in capabilities surveillance workforce
☐ Other, please specify

29. Are there any workforce development initiatives in place or under development to address these limitations?

☐ Yes - led by the National Public Health Institute
☐ Yes - led by the Ministry of Health
☐ Yes - led by academic institutions within the country
☐ Yes - led by private industry

☐ Yes - externally funded / supported
☐ No
☐ Other, please specify

Integrated Disease Surveillance (IDS)

30. Integrated disease surveillance can be described as "a combination of active and passive systems using a single infrastructure that gathers information about multiple diseases or behaviours of interest [to ensure robust early warning and a prompt public health response]." (Nsubuga et al., 2003).

    According to this definition, does your country have an integrated diseases surveillance (IDS) system in place?*

    -- Please Select --

Questions for countries which have an Integrated Disease Surveillance system in place

Integration of Surveillance Systems

In the following five questions, we will ask you about core functions of your IDS system. If multiple programs or mechanisms feed into your IDS, please consider the functionality of the IDS on average / a system wide basis.

31. How well established is your IDS system's ability to **detect and report events / indicators**?

○ National strategy, guidelines and/or SOPs for surveillance are not available or under development
○ National strategy, guidelines and/or SOPs for surveillance have been developed but not implemented. The surveillance system is functioning but lacks systematic immediate reporting or weekly reporting of events and/or data
○ National strategy, guidelines and/or SOPs for surveillance have been developed and are being implemented at the national level. The surveillance system provides immediate and weekly reporting of events and/or data with lab results integrated
○ National strategy, guidelines and/or SOPs for surveillance have been developed and are being implemented at the national and intermediate levels. The surveillance system provides immediate and weekly reporting of events and/or data with lab results integrated and integration between IBS and EBS
○ National strategy, guidelines and/or SOPs for surveillance linking all sectors have been developed and implemented at national, intermediate and primary public health levels; and the system is exercised (as applicable), reviewed, evaluated and updated on a regular basis, with improvement at all levels in the country, with all components linked to one national surveillance system

32. How well established is your IDS system's ability to **investigate and/or verify events**?

○ Method, process or mechanisms for verifying and investigating detected events is not available or under development
○ Method, process or mechanisms for verifying and investigating detected events has been developed but not implemented
○ Method, process or mechanisms for verifying and investigating detected events has been developed and is being implemented at the national and intermediate level
○ Method, process or mechanisms for verifying, investigating and risk assessing detected events has been developed and is being implemented at the national and intermediate levels, involving trained personnel from multiple sectors
○ Method, process or mechanisms for verifying, investigating and risk assessing detected events is being implemented at national, intermediate and primary public health levels, involving trained personnel from multiple sectors and exercised (as applicable), reviewed, evaluated and updated on a regular basis

33. How well established is your IDS system's ability to **analyse data and report results**?

○ Surveillance data are received sporadically and analysed on some priority diseases, or unusual events, often with delay
○ Surveillance data are received regularly (i.e., weekly and/or monthly). An ad hoc team does some analysis of data for some priority diseases, or unusual events
○ Surveillance data are received regularly and analysed on some priority diseases, or unusual events, often with delay. Data is shared across sectors
○ Surveillance data are received and analysed regularly. Epidemiological

bulletins are generated and disseminated across sectors and internationally on regular basis. Data is shared across sectors and internationally on a regular basis
○Surveillance data analysis is conducted, and epidemiological bulletins are generated and disseminated across sectors and internationally on regular basis. An electronic platform and a dedicated team support data management and generation of epidemiological bulletins. Data is shared across sectors and internationally on a regular basis. Capacity for advanced data analysis is supported and ensured

34. How well established is your IDS system's ability to **respond to a public health event**?

○National strategy, guidelines and/or SOPs for IDS in response to a public health event are not available or underdevelopment
○National strategy, guidelines and/or SOPs for IDS in response to a public health event have been developed but not implemented
○IDS system produces standard data for use in response at a national level
○IDS system has capacity to adapt or expand data collection and reporting in response to a public health event. Data for response are available at a national and intermediate level.
○IDS system has mechansims in place to quickly to adapt or expand data collection and reporting in response to a public health event. Surveillance data are routinely used in forecasting. Data for response are available at a national, intermediate, and primary public health levels.

35. How well established is your IDS system's ability to **evaluate and provide feedback for system improvement**?

○National strategy, guidelines and/or SOPs for IDS system monitoring and evaluation are not available or underdevelopment
○National strategy, guidelines and/or SOPs for IDS system monitoring and evaluation have been developed but not implemented
○IDS system evaluation conducted and/or monitoring data reviewed at a national level at least once a year
○IDS system evaluation conducted and/or monitoring data reviewed at a national level and intermediate level multiple times per year
○IDS system evaluation conducted, and monitoring data reviewed at a national level and intermediate levels multiple times per year

36. What challenges have you experienced in setting up and running your IDS system? Tick all that apply.

To note, we are keen to identify case studies describing innovative or best practice in the development of IDS systems, and overcoming the challenges which that may present. These questions can be found towards the end of this survey.

☐Governance: leadership, accountability, regulation and enforcement
☐Financial: inadequate investment, multi-year budget not available
☐Data availability: requisite data not collected, not collected to a suitably high standard, or not shared by the organisations who are responsible for collecting that data
☐Data sharing and ownership: lack of involvement, unclear roles and responsibilities, internal politics, unclear lines of reporting and accountability, territorialism, conflict / uncertainty re: intended use of data
☐IT systems interoperability: incompatible IT systems to migrate data, migration from paper to electronic format, suboptimal IT systems, data security, data protection
☐Analysis and reporting: lack of statistical package, unavailability of big data analysis
☐Laboratory: lack of testing capabilities, lack of multi-sectoral reporting, lack of provider reporting
☐Workforce capacity and capabilities: lack of experience and skills in multi-sectoral working, lack of analytical skills, lack of data collection skills
☐Other, please specify

37. Semantic consistency in terms of surveillance is the concept of “providing access to data and minimizing the potential for errors in human interpretation through the creation of standard data definitions and formats” (George et al., 2020)

To what extent is the integration of the different areas of your surveillance

system supported by 'semantic consistency?'

○ Not at all – different surveillance systems use different languages, data definitions and formats. These are a barrier to greater integration.
○ To some extent – a number of surveillance systems operate use standardised language, data definitions and formats to support their integration. However, this is not the case across all surveillance systems, and there is more work to do in this regard.
○ To a great extent – almost all surveillance systems operate using a standardised language, data definitions and formats which supports integration across the system.

38. Are there any specific surveillance systems or data sources from the sectors contributing to your surveillance system that cannot be integrated with existing systems, due to lack of interoperability, data sharing, or another reason?

☐ Public healthcare providers
☐ Private healthcare providers
☐ Public health
☐ Veterinarian and animal health services
☐ Agriculture sector
☐ Environmental sector
☐ Laboratory
☐ Private sector
☐ Communities
☐ Pharmaceutical sector
☐ Occupational health
☐ Chemical and poison sector
☐ Disaster management
☐ Food safety sector
☐ Biosafety and biosecurity
☐ Other, please specify

39. Are there any specific surveillance systems or data sources from **disease-specific programmes in your country** that are not integrated with existing systems, due to lack of interoperability, data sharing, or another reason?

☐ HIV / AIDS
☐ Malaria
☐ Tuberculosis (TB)
☐ Cholera
☐ Measles
☐ COVID-19
☐ Non-communicable diseases (NCDs)
☐ Other, please specify

40. Are there any specific surveillance systems or data sources from **specific agency or organisation working in your country** that cannot be integrated with existing systems, due to lack of interoperability, data sharing, or another reason?

☐ Academic institution (please specify below)
☐ Government ministry or agency (please specify below)
☐ Non-governmental organization (please specify below)
☐ Please provide details related to the answers you have provided above.

41. Are there any specific surveillance systems or data sources from **specific surveillance platforms or data sources** that cannot be integrated with existing systems, due to lack of interoperability, data sharing, or another reason?

☐ Notifiable disease surveillance systems
☐ Syndromic surveillance
☐ Sentinel surveillance
☐ Laboratory based surveillance

☐ Health facility based surveillance
☐ Hospital-based surveillance (e.g. admissions, discharges, emergency unit)
☐ Case-based surveillance
☐ Disease-specific surveillance
☐ Community-based surveillance
☐ Mortality surveillance
☐ Genomic surveillance
☐ Behavioural surveillance
☐ Vaccine surveillance (uptake / effectiveness / side effects)
☐ Routine health indicators surveillance
☐ Surveys and research
☐ Mortality surveillance
☐ Health and demographic surveillance system (e.g. using civil registration and vital statistics (CRVS))
☐ Environmental pathogen surveillance (e.g. sewage)
☐ Other, please specify

42. What is the reason the surveillance data, described in the questions above, are not integrated?

If more than one sector / program / agency / platform selected, please specify the reason for each selection.

Ownership and responsibility for the Integrated Disease Surveillance

43. Is the National Public Health Institute responsible the Integrated Disease Surveillance system?

○ Yes, sole owner
○ Yes, joint owner
○ No

44. Who is responsible for the Integrated Disease Surveillance system?
☐ Ministry of Health
☐ Center for Statistics
☐ Other, please specify

45. Who is jointly responsible for the Integrated Disease Surveillance system, alongside the NPHI?
☐ Ministry of Health
☐ Center for Statistics
☐ Other, please specify

46. Who is the lead agency responsible for each of the core functions related to the Integrated Disease Surveillance system? Please select all that apply.

	NPHI	MOH	Center for Statistics	No clear lead	Other, please specify
Case/event detection	☐	☐	☐	☐	☐
Case/event reporting	☐	☐	☐	☐	☐
Analysis	☐	☐	☐	☐	☐
Investigation or confirmation	☐	☐	☐	☐	☐
Response	☐	☐	☐	☐	☐
Feedback	☐	☐	☐	☐	☐
Evaluation	☐	☐	☐	☐	☐
Preparedness	☐	☐	☐	☐	☐

47. If you selected 'other' above, please provide details below.

Lab support for the Integrated Disease Surveillance system

48. From which laboratories are data reported into the Integrated Disease Surveillance system?

- ▢ National public health laboratories
- ▢ Sub-national public health laboratories
- ▢ Other public sector laboratories
- ▢ Private sector laboratories
- ▢ Regional supranational laboratories
- ▢ Other, please specify

49. How are lab results integrated into the Integrated Disease Surveillance system?

- ▢ Electronically through compatible IT systems
- ▢ Electronically other (please specify below)
- ▢ Manually (please specify below)
- ▢ Lab results not yet integrated into the Integrated Disease Surveillance system
- ▢ Other, please specify

50. Is genomic testing / sequencing available for the Integrated Disease Surveillance system specimens?

- ▢ Yes, national public health laboratories
- ▢ Yes, sub-national public health laboratories
- ▢ Yes, private sector laboratories
- ▢ Yes, outside of country
- ▢ No

51. What are the barriers to the effective integration of lab data in to the Integrated Disease Surveillance system?

- ▢ Limited staff
- ▢ Lack of equipment/supplies
- ▢ Inefficient specimen transfer
- ▢ Poor data systems/integration
- ▢ No barriers to report
- ▢ Other, please specify

External support for the Integrated Disease Surveillance system

52. Are you engaging with international partners and expertise from outside of your country in running and improving your Integrated Disease Surveillance system?

- ▢ Yes, private sector/consultancy
- ▢ Yes, international National Public Health Institute
- ▢ Yes, international governmental organisations
- ▢ Yes, non-governmental organizations
- ▢ Yes, regional public health agencies
- ▢ Yes, other (please specify below)
- ▢ No
- ▢ Other, please specify

Financing

53. How is your Integrated Disease surveillance financed? (tick all that apply)

- ▢ National government
- ▢ NGO/Philanthropic
- ▢ Private
- ▢ International aid funding from other country partner
- ▢ International aid funding from a non-government organization
- ▢ Other, please specify

54. If "Other", please elaborate.

55. Is this funding time-limited / one-off or multi-year dedicated funding?
- ▢ Time limited
- ▢ Multi-year funding
- ▢ Other, please specify

56. If time-limited, how long is Integrated Disease Surveillance system funding available for? (answer in months)

Public Health Institutes of the World
IANPHI

Questions for countries which have an Integrated Disease Surveillance system in development

Integration of Surveillance Systems

In the following five questions, we will ask you about core functions of the IDS system your country is developing. If multiple programs or mechanisms feed into your IDS, please consider the functionality of the IDS on average / a system wide basis.

57. How well established is your IDS system's ability to detect and report events / indicators? Please answer with regard to the stage at which your IDS is currently developed.

- ○ National strategy, guidelines and/or SOPs for surveillance are not available or under development
- ○ National strategy, guidelines and/or SOPs for surveillance have been developed but not implemented. The surveillance system is functioning but lacks systematic immediate reporting or weekly reporting of events and/or data
- ○ National strategy, guidelines and/or SOPs for surveillance have been developed and are being implemented at the national level. The surveillance system provides immediate and weekly reporting of events and/or data with lab results integrated
- ○ National strategy, guidelines and/or SOPs for surveillance have been developed and are being implemented at the national and intermediate levels. The surveillance system provides immediate and weekly reporting of events and/or data with lab results integrated and integration between IBS and EBS
- ○ National strategy, guidelines and/or SOPs for surveillance linking all sectors have been developed and implemented at national, intermediate and primary public health levels; and the system is exercised (as applicable), reviewed, evaluated and updated on a regular basis, with improvement at all levels in the country, with all components linked to one national surveillance system

58. How well established is your IDS system's ability to **investigate and/or verify events**?

Please answer with regard to the stage at which your IDS is currently developed.

- ○ Method, process or mechanisms for verifying and investigating detected events is not available or under development
- ○ Method, process or mechanisms for verifying and investigating detected events has been developed but not implemented
- ○ Method, process or mechanisms for verifying and investigating detected events has been developed and is being implemented at the national and intermediate level
- ○ Method, process or mechanisms for verifying, investigating and risk assessing detected events has been developed and is being implemented at the national and intermediate levels, involving trained personnel from multiple sectors
- ○ Method, process or mechanisms for verifying, investigating and risk assessing detected events is being implemented at national, intermediate and primary public health levels, involving trained personnel from multiple sectors and exercised (as applicable), reviewed, evaluated and updated on a regular basis

59. How well established is your IDS system's ability to **analyze data and report results**?

Please answer with regard to the stage at which your IDS is currently developed.

○Surveillance data are received sporadically and analysed on some priority diseases, or unusual events, often with delay
○Surveillance data are received regularly (i.e., weekly and/or monthly). An adhoc team does some analysis of data for some priority diseases, or unusual events
○Surveillance data aSurveillance data are received regularly and analysed for some priority diseases, or unusual events. Data and ad hoc reports may be shared across sectors
○Surveillance data are received and analysed regularly for most priority diseases and unusual events. Epidemiological bulletins are generated and disseminated across sectors and internationally on regular basis. Data is shared across sectors and internationally on a regular basis
○Surveillance data are received and analysed regularly for all priority diseases and unusual events. Epidemiological bulletins are generated and disseminated across sectors and internationally on regular basis. An electronic platform and a dedicated team support data management and generation of epidemiological bulletins. Data is shared across sectors and internationally on a regular basis. Capacity for advanced data analysis is supported and ensured.

60. How well established is your IDS system's ability to **respond to a public health event**?

Please answer with regard to the stage at which your IDS is currently developed.

○National strategy, guidelines and/or SOPs for IDS in response to a public health event are not available or underdevelopment
○National strategy, guidelines and/or SOPs for IDS in response to a public health event have been developed but not implemented
○IDS system produces standard data for use in response at a national level
○IDS system has capacity to adapt or expand data collection and reporting in response to a public health event. Data for response are available at a national and intermediate level.
○IDS system has mechansims in place to quickly to adapt or expand data collection and reporting in response to a public health event. Surveillance data are routinely used in forecasting. Data for response are available at a national, intermediate, and primary public health levels.

61. How well established is your IDS system's ability to **evaluate and provide feedback for system improvement**?

Please answer with regard to the stage at which your IDS is currently developed.

○National strategy, guidelines and/or SOPs for IDS system monitoring and evaluation are not available or underdevelopment
○National strategy, guidelines and/or SOPs for IDS system monitoring and evaluation have been developed but not implemented
○IDS system evaluation conducted and/or monitoring data reviewed at a national level at least once a year
○IDS system evaluation conducted and/or monitoring data reviewed at a national level and intermediate level multiple times per year
○IDS system monitoring and evaluation conducted continuously at a national, intermediate, and primary public health level and routinely applied for system strengthening

62. What challenges have you experienced in setting up your IDS system? (tick all that apply)

To note, we are keen to identify case studies describing innovative or best practice in the development of IDS systems, and overcoming the challenges which that may present. These questions can be found towards the end of this survey.

☐Governance: leadership, accountability, regulation and enforcement
☐Financial: inadequate investment, multi-year budget not available
☐Data availability: requisite data not collected, not colleted to a suitably high standard, or not shared by the organisations who are responsible for collecting that data
☐Data sharing and ownership: lack of involvement, unclear roles and responsibilities, internal politics, unclear lines of reporting and accountability, territorialism, conflict / uncertainty re: intended use of data
☐IT systems: incompatible IT systems to migrate data, migration from paper to electronic format, suboptimal IT systems, lack of data security or data protection Analysis and reporting: lack of statistical and computing resources, lack of analytical capabilities
☐Analysis and reporting: lack of statistical package, unavailability of big

data analysis
☐ Laboratory: lack of testing capabilities, lack of multi-sectoral reporting, lack of provider reporting
☐ Workforce capacity and capabilities: lack of experience and skills in multi-sectoral working, lack of analytical skills, lack of data collection skills
☐ Other, please specify

63. Semantic consistency in terms of surveillance is the concept of "providing access to data and minimizing the potential for errors in human interpretation through the creation of standard data definitions and formats" (George et al., 2020)

To what extent is the integration of the different areas of your surveillance system supported by 'semantic consistency?'
○ Not at all – different surveillance systems use different languages, data definitions and formats. These are a barrier to greater integration.
○ To some extent – a number of surveillance systems operate use standardised language, data definitions and formats to support their integration. However, this is not the case across all surveillance systems, and there is more work to do in this regard.
○ To a great extent – almost all surveillance systems operate using a standardised language, data definitions and formats which supports integration across the system.

64. Are there any specific surveillance systems or data sources from the sectors contributing to your surveillance system that are not integrated with existing systems, due to lack of interoperability, data sharing, or another reason?

☐ Public healthcare providers
☐ Private healthcare providers
☐ Public health
☐ Veterinarian and animal health services
☐ Agriculture sector
☐ Environmental sector
☐ Laboratory
☐ Private sector
☐ Communities
☐ Pharmaceutical sector
☐ Occupational health
☐ Chemical and poison sector
☐ Disaster management
☐ Food safety sector
☐ Biosafety and biosecurity
☐ Other, please specify

65. Are there any specific surveillance systems or data sources from **disease-specific programmes in your country** that are not integrated with existing systems, due to lack of interoperability, data sharing, or another reason?
☐ HIV / AIDS
☐ Malaria
☐ Tuberculosis (TB)
☐ Cholera
☐ Measles
☐ COVID-19
☐ Non-Communicable Diseases (NCDs)
☐ Other, please specify

66. Are there any specific surveillance systems or data sources from **specific agency or organisation working in your country** that are not integrated with existing systems, due to lack of interoperability, data sharing, or another reason?
☐ Academic institution (please specify below)
☐ Government ministry or agency (please specify below)
☐ Non-governmental organization (please specify below)

☐ Please provide details related to the answers you have provided above.

67. Are there any specific surveillance systems or data sources from **specific surveillance platforms or data sources** that cannot be integrated with existing systems, due to lack of interoperability, data sharing, or another reason?

☐ Notifiable disease surveillance systems
☐ Syndromic surveillance
☐ Sentinel surveillance
☐ Laboratory based surveillance
☐ Health facility based surveillance
☐ Hospital-based surveillance (e.g. admissions, discharges, emergency unit)
☐ Case-based surveillance
☐ Disease-specific surveillance
☐ Community-based surveillance
☐ Mortality surveillance
☐ Genomic surveillance
☐ Behavioural surveillance
☐ Vaccine surveillance (uptake / effectiveness / side effects)
☐ Routine health indicators surveillance
☐ Surveys and research
☐ Mortality surveillance
☐ Health and demographic surveillance system (e.g. using civil registration and vital statistics (CRVS))
☐ Environmental pathogen surveillance (e.g. sewage)
☐ Other, please specify

68. What is the reason the surveillance data, as described in the questions above, are not integrated?

If more than one sector / program / agency / platform selected, please specify the reason for each selection.

Ownership of Integrated Disease Surveillance

69. Is there a national IDS plan/policy in place?

○ Yes
○ No
○ Unknown

70. Has this plan been implemented?

○ Yes
○ No
○ Unknown

71. If yes, at what level?

Multiple options allowed

☐ National level
☐ Subnational level
☐ Peripheral level
☐ Other, please specify

72. Once fully in place, will the National Public Health Institute be the owner of Integrated Disease Surveillance system?

○ Yes, sole owner
○ Yes, joint owner
○ No

73. Who will be the owner of the Integrated Disease Surveillance system once it is fully implemented?

☐ Ministry of Health
☐ Center for Statistics

☐ Other, please specify

74. Who will be the joint owner/s of the Integrated Disease Surveillance system once it is fully implemented, alongside the NPHI?

☐ Ministry of Health
☐ Center for Statistics
☐ Other, please specify

75. Is there an approved organisational plan for an Integrated Disease Surveillance system to be implemented by the lead agency/agencies?

○ Yes
○ No
○ Unknown

76. Who will be responsible for each of the core functions related to the Integrated Disease Surveillance system? Select all that apply.

	NPHI	MOH	Center for Statistics	No clear lead	Other, specify below
Case/event detection	☐	☐	☐	☐	☐
Case/event reporting	☐	☐	☐	☐	☐
Analysis	☐	☐	☐	☐	☐
Investigation or confirmation	☐	☐	☐	☐	☐
Response	☐	☐	☐	☐	☐
Feedback	☐	☐	☐	☐	☐
Evaluation	☐	☐	☐	☐	☐
Preparedness	☐	☐	☐	☐	☐

77. Other, please specify

Lab support for the Integrated Disease Surveillance system

78. What organization(s) provide laboratory support for the Integrated Disease Surveillance system?

☐ National public health laboratories
☐ Sub-national public health laboratories
☐ Other public sector laboratories
☐ Private sector laboratories
☐ Regional supranational laboratories
☐ Other, please specify

79. How are lab results integrated into the Integrated Disease Surveillance system?

☐ Electronically through compatible IT systems
☐ Electronically other (please specify below)
☐ Manually (please specify below)
☐ Lab results not yet integrated into the Integrated Disease Surveillance system
☐ Other, please specify

80. Is genomic testing / sequencing available for the Integrated Disease Surveillance system specimens?

☐ Yes, national public health laboratories
☐ Yes, sub-national public health laboratories
☐ Yes, private sector laboratories
☐ Yes, outside of country
☐ No

☐ Other, please specify

81. What are the barriers to the effective integration of lab data in to the Integrated Disease Surveillance system?
   - ☐ Limited staff
   - ☐ Lack of equipment/supplies
   - ☐ Inefficient specimen transfer
   - ☐ Poor data systems/integration
   - ☐ No barriers to report
   - ☐ Other, please specify

External support for the Integrated Disease Surveillance system

82. Are you engaging with international partners and expertise from outside of your country in running and improving your Integrated Disease Surveillance system?
   - ☐ Yes, private sector/consultancy
   - ☐ Yes, international National Public Health Institute
   - ☐ Yes, international governmental organisations
   - ☐ Yes, non-governmental organizations
   - ☐ Yes, regional public health agencies
   - ☐ Yes, other (please specify below)
   - ☐ No
   - ☐ Other, please specify

Financing

83. Do you have funding in place for your IDS system?
   - ○ Yes
   - ○ No

84. How is your Integrated Disease surveillance financed? (tick all that apply)
   - ☐ National government
   - ☐ NGO/Philanthropic
   - ☐ Private
   - ☐ International aid funding from other country partner
   - ☐ International aid funding from a non-government organization
   - ☐ Other, please specify

85. If "Other", please elaborate.

86. Is this funding time-limited / one-off or multi-year dedicated funding?
   - ○ Time limited
   - ○ Multi-year funding
   - ○ Other, please specify

87. If time-limited, how long is Integrated Disease Surveillance system funding available for? (answer in months)

88. If there is currently no funding, how do you plan to finance your Integrated Disease Surveillance system?
   - ☐ National government
   - ☐ NGO/Philanthropic
   - ☐ Private
   - ☐ International aid funding from other country partner
   - ☐ International aid funding from a non-government organization
   - ☐ Other, please elaborate

Public Health Institutes of the World
IANPHI

Questions for countries which do not have an Integrated Disease Surveillance system

Integration of Surveillance Systems

89. What has prevented your country establishing an IDS system? (Tick all that apply)

☐ Governance: leadership, accountability, regulation and enforcement
☐ Financial: inadequate investment, multi-year budget not available
☐ Data availability: requisite data not collected, not colleted to a suitably high standard, or not shared by the organisations who are responsible for collecting that data
☐ Data sharing and ownership: lack of involvement, unclear roles and responsibilities, internal politics, unclear lines of reporting and accountability, territorialism, conflict / uncertainty re: intended use of data
☐ IT systems: incompatible IT systems to migrate data, migration from paper to electronic format, suboptimal IT systems, data security, data protection
☐ Analysis and reporting: lack of statistical package, unavailability of big data analysis,
☐ Laboratory: lack of testing capabilities, lack of multi-sectoral reporting, lack of provider reporting
☐ Capacity and capabilities: lack of multi-sectoral training, lack of analytical skills, lack of data collection skills
☐ Other, please specify

90. Is there a national Integrated Disease Surveillance system plan / policy in development?

○ Yes
○ No
○ Unknown
○ Other, please specify

91. Semantic consistency in terms of surveillance is the concept of "providing access to data and minimizing the potential for errors in human interpretation through the creation of standard data definitions and formats" (George et al., 2020)

Though there is no IDS in place in your country at present, to what extent is the integration of the different areas of your surveillance system supported by 'semantic consistency?'

○ Not at all – different surveillance systems use different languages, data definitions and formats. These are a barrier to greater integration.
○ To some extent – a number of surveillance systems operate use standardised language, data definitions and formats to support their integration. However, this is not the case across all surveillance systems, and there is more work to do in this regard.
○ To a great extent – almost all surveillance systems operate using a standardised language, data definitions and formats which supports integration across the system.

Support and funding for the development of an IDS

92. Are you engaging with international partners and expertise from outside of your country in developing plans for a future Integrated Disease Surveillance system?

- ▢ Yes - private sector/consultancy
- ▢ Yes - international national public health institute
- ▢ Yes - international governmental organisations
- ▢ Yes - non-governmental organisations
- ▢ Yes - regional public health agencies
- ▢ No
- ▢ Yes - other, please specify

93. Are you exploring potential sources of funding with a view to developing an Integrated Disease Surveillance system?

- ○ Yes
- ○ No
- ○ Unknown

94. If yes, which sources of funding are you exploring?

- ▢ National government
- ▢ NGO/Philanthropic
- ▢ Private
- ▢ International aid funding from other country partner
- ▢ International aid funding from a non-government organization
- ▢ Other, please specify

Public Health Institutes of the World
IANPHI

Future development of surveillance systems

95. What are the current areas of focus in the development of your surveillance systems? Please rate each category as follows:

- **Not a priority:** little to no attention, time or resources is being committed to the development of this area of surveillance in my country
- **A priority:** some attention, time or resources is being committed to the development of this area of surveillance in my country
- **A high priority:** a lot of attention, time or resources is being committed to the development of this area of surveillance in my country

	Not a priority	A priority	A high priority
Greater cross-border sharing of surveillance data with other countries and across the region.	○	○	○
Greater integration of surveillance systems at the national level.	○	○	○
Greater integration of surveillance systems at the sub-national level.	○	○	○
Training and development of the workforce to support surveillance.	○	○	○
Development of technical guidelines for integration of surveillance data.	○	○	○
Monitoring and evaluation of surveillance systems to improve performance	○	○	○
Situational analysis to identify gaps in the surveillance system	○	○	○
Securing access to data collected by public sector / governmental organisations that is not currently collected by NPHI / the relevant public health authority	○	○	○
Securing access to data collected by private sector and nongovernmental organisations that is not currently collected by NPHI / the relevant public health authority	○	○	○
Securing legislative / legal mandate to collect surveillance data needed for planning, preparedness and response work	○	○	○

Developing timely and transparent reporting systems for the surveillance data that is collected.	○	○	○
Securing funding for the development of surveillance systems.	○	○	○
Securing political support for the further development of surveillance systems	○	○	○
Development of the requisite IT and digital infrastructure and tools needed to support surveillance systems.	○	○	○
Increasing interconnectedness and interoperability of surveillance systems.	○	○	○
Increasing access to and capacity of laboratory services to support surveillance systems.	○	○	○
Improving access to genomic surveillance tools	○	○	○
Improving data transparency, to ensure the visibility of all national threats by NPHIs and by WHO for transnational threats	○	○	○

96. To provide further details related to the current areas of focus in the development of your surveillance systems, please use the box below.

Response to COVID-19

97. Covid-19 presented significant challenges to countries' health systems, including surveillance. However they also identified ways in which surveillance and other parts of the health system might be improved to prevent, detect and respond to future outbreaks.

We are interested in hearing about your experience responding to Covid-19 and whether it accelerated or supported the implementation of aspects of your surveillance as part of your country's Covid-19 response.

Please use the text box below to provide examples? Please outline whether the changes to your surveillance system brought about by Covid-19 are continuing or were time-limited.

98. How did your surveillance system facilitate or make your response to COVID easier or more effective?

Integration of surveillance systems: examples of innovative practice

99. We would like to identify areas of innovative practice related to surveillance

integration and IDS and to highlight these in the report IANPHI will produce using the data captured in this survey.

Examples could include the development of communities of practice, the adoption of technology, data sharing and analytics, capacity and capabilities, integration of communicable and non-communicable diseases, One Health integration, data analytics for reducing inequities, evaluation of interventions, multi-sectoral engagement and governance etc.

Please provide information of such examples in the text box below. We will contact you beforehand for further information and to seek permission to use these examples in our report.

IANPHI Integrated Disease Surveillance Country Survey - English

Page 9

Public Health Institutes of the World
IANPHI

100. Integrated Disease Surveillance has been described as 'a combination of active and passive systems using a single infrastructure that gathers information about multiple diseases or behaviours of interest' (Nsubuga et al., 2006).

How well does this definition describe Integrated Disease Surveillance?

- ○ Not at all well - it misses important elements of IDS
- ○ Somewhat - it is a good definition though does not describe IDS in its totality
- ○ Very well - the definition completely captures all elements of ID

101. Based on your experience, including the experience of your country context and needs, what else, if anything, would you add to the Nsubuga et al. definition provided above?

102. The table presented below is from a paper published in the Lancet titled 'Disease surveillance for the COVID-19era: time for bold changes' (Morgan et al., 2021). The authors state that Integrated Disease Surveillance should be underpinned by the five principles they set out: population based, laboratory confirmation, digital data, data transparency and adequate financing.

In your experience, given your country context and needs, do you think there are other principles for IDS that should be incorporated to those five principles set out in the table below?

	Benefits	Implementation requirement
Population-based	Denominators for mortality rates and disease burden	CRVS or sample registration system
Laboratory confirmation	Cases accurately tracked	Capacity to scale testing and sequence pathogens
Digital data	Systems interconnected and privacy protected	Unique health identifiers, standard metadata, web accessible
Data transparency	Visibility of all national threats by NPHIs and by WHO for transnational threats	Automated reporting to NPHI with a subset to WHO and regional bodies
Adequate financing	Sustainable country-owned systems	Invest US$1–4 per capita annually

CRVS=civil registration and vital statistics. NPHI=national public health institute.

***Table:* Core principles for integrated disease surveillance**

103. Is there anything that you would like to add with regard to surveillance systems and integration that you have had the opportunity to include in this survey?

Please use the text box below to include any concluding thoughts.
