# Supplementary material for: Surveillance system integration: reporting the results of a global multicountry survey
Source: Public Health. Author manuscript; Available in PMC 2024 Aug 1. (PMC11292781; doi:10.1016/j.puhe.2024.03.004)
Supplement: Supplementary Files [file NIHMS2009509-supplement-Supplementary_Files.zip › 1-s2.0-S0033350624001069-mmc1.docx]

**Supplementary Material**

In addition to Figures 1 and 2 below, the supplementary tables (1 to 30) provided in this document set out the aggregated and anonymised results from the IDS survey.

**Supplementary Figure 1. IDS Conceptual Model**

**
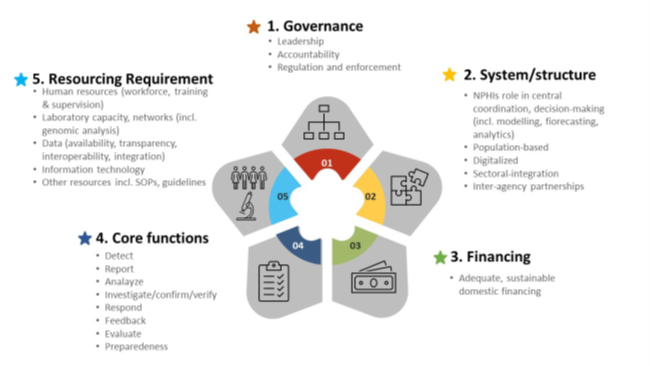
**

**Supplementary Figure 2. Core functions of IDS by World Bank income groups**

| **a) High-income** (Developed n = 4; Partial n = 13) | | | | | | |
| --- | --- | --- | --- | --- | --- | --- |
| **Core** | **Weak** | **Moderate-weak** | **Moderate** | **Moderate-strong** | **Strong** | **Total** |
| **functions** |  |  |  |  |  |  |
| *Detect* | 0% | 24% | 12% | 59% | 6% | 100% |
| *Investigate* | 6% | 12% | 24% | 41% | 18% | 100% |
| *Analyse* | 0% | 0% | 24% | 41% | 35% | 100% |
| *Respond* | 6% | 0% | 12% | 65% | 18% | 100% |
| *Evaluate and provide feedback* | 20% | 33% | 27% | 7% | 13% | 100% |
|  |  |  |  |  |  |  |
| **b) Upper middle-income** (Developed n = 2; Partial n = 11) | | | | | | |
| **Core** | **Weak** | **Moderate-weak** | **Moderate** | **Moderate-strong** | **Strong** | **Total** |
| **functions** |  |  |  |  |  |  |
| *Detect* | 15% | 8% | 23% | 31% | 23% | 100% |
| *Investigate* | 0% | 0% | 38% | 31% | 31% | 100% |
| *Analyse* | 8% | 8% | 8% | 54% | 23% | 100% |
| *Respond* | 8% | 8% | 0% | 54% | 31% | 100% |
| *Evaluate and provide feedback* | 23% | 23% | 23% | 15% | 15% | 100% |
|  |  |  |  |  |  |  |
| **c) Lower middle-income** (Developed n = 5; Partial n = 6) | | | | | | |
| **Core** | **Weak** | **Moderate-weak** | **Moderate** | **Moderate-strong** | **Strong** | **Total** |
| **functions** |  |  |  |  |  |  |
| *Detect* | 9% | 18% | 9% | 27% | 36% | 100% |
| *Investigate* | 9% | 18% | 18% | 9% | 45% | 100% |
| *Analyse* | 18% | 9% | 27% | 27% | 18% | 100% |
| *Respond* | 18% | 0% | 9% | 45% | 27% | 100% |
| *Evaluate and provide feedback* | 18% | 9% | 45% | 0% | 27% | 100% |
|  |  |  |  |  |  |  |
| **d) Low-income** (Developed n = 5*; Partial n = 4) | | | | | | |
| **Core** | **Weak** | **Moderate-weak** | **Moderate** | **Moderate-strong** | **Strong** | **Total** |
| **functions** |  |  |  |  |  |  |
| *Detect* | 11% | 11% | 22% | 56% | 0% | 100% |
| *Investigate* | 10% | 20% | 20% | 50% | 0% | 100% |
| *Analyse* | 10% | 20% | 20% | 40% | 10% | 100% |
| *Respond* | 10% | 10% | 20% | 40% | 20% | 100% |
| *Evaluate and provide feedback* | 11% | 22% | 44% | 22% | 0% | 100% |
| *There were only 4 responses from developed IDS systems for the “*evaluate and provide feedback*” core function | | | | | | |
| ** Proportions may not total 100 due to rounding | | | | | | |

**Supplementary Table 1. Surveillance systems being implemented in country**

| **Surveillance System** | **Overall** | **IDS Status** | | | **WB Income Group** | | | |
| --- | --- | --- | --- | --- | --- | --- | --- | --- |
| **n/N ( % )** |  | **Developed IDS** | **Partial IDS** | **No IDS** | **Low** | **Lower-middle** | **Upper-middle** | **High** |
| Notifiable Diseases | 64/64 (100%) | 16/16 (100%) | 36/36 (100%) | 12/12 (100%) | 11/11 (100%) | 14/14 (100%) | 15/15 (100%) | 23/23 (100%) |
| Syndromic Surveillance | 62/65 (95%) | 16/16 (100%) | 34/36 (94%) | 12/13 (92%) | 11/11 (100%) | 15/15 (100%) | 14/15 (93%) | 21/23 (91%) |
| Sentinel Surveillance | 63/63 (100%) | 16/16 (100%) | 35/35 (100%) | 12/12 (100%) | 11/11 (100%) | 13/13 (100%) | 15/15 (100%) | 23/23 (100%) |
| Lab-based Surveillance | 63/64 (98%) | 16/16 (100%) | 35/35 (100%) | 12/13 (92%) | 11/11 (100%) | 13/14 (93%) | 15/15 (100%) | 23/23 (100%) |
| Hospital-based Surveillance | 62/64 (97%) | 15/16 (94%) | 34/35 (97%) | 13/13 (100%) | 11/11 (100%) | 14/14 (100%) | 15/15 (100%) | 22/23 (96%) |
| Other Facility-based Surveillance | 59/60 (98%) | 16/16 (100%) | 31/32 (97%) | 12/12 (100%) | 11/11 (100%) | 13/13 (100%) | 14/14 (100%) | 20/21 (95%) |
| Case-based Surveillance | 61/62 (98%) | 16/16 (100%) | 33/34 (97%) | 12/12 (100%) | 11/11 (100%) | 13/13 (100%) | 14/14 (100%) | 23/23 (100%) |
| Disease-specific Surveillance | 64/64 (100%) | 16/16 (100%) | 36/36 (100%) | 12/12 (100%) | 11/11 (100%) | 14/14 (100%) | 15/15 (100%) | 23/23 (100%) |
| Community-based Surveillance | 46/60 (77%) | 15/16 (94%) | 24/32 (75%) | 7/12 (58%) | 11/11 (100%) | 10/13 (77%) | 10/15 (67%) | 14/20 (70%) |
| Mortality Surveillance | 60/65 (92%) | 14/16 (88%) | 35/36 (97%) | 11/13 (85%) | 9/11 (82%) | 12/15 (80%) | 15/15 (100%) | 23/23 (100%) |
| Genomic Surveillance | 57/64 (89%) | 16/16 (100%) | 30/35 (86%) | 11/13 (85%) | 9/11 (82%) | 13/15 (87%) | 11/14 (79%) | 23/23 (100%) |
| Behavioral Surveillance | 35/56 (62%) | 9/13 (69%) | 21/33 (64%) | 5/10 (50%) | 5/9 (56%) | 6/11 (55%) | 7/14 (50%) | 17/22 (77%) |
| Vaccination Coverage | 64/64 (100%) | 16/16 (100%) | 36/36 (100%) | 12/12 (100%) | 11/11 (100%) | 14/14 (100%) | 15/15 (100%) | 23/23 (100%) |
| Vaccine Effectiveness | 59/63 (94%) | 15/16 (94%) | 33/35 (94%) | 11/12 (92%) | 10/11 (91%) | 12/14 (86%) | 13/14 (93%) | 23/23 (100%) |
| Survey and Research | 49/58 (84%) | 15/16 (94%) | 27/32 (84%) | 7/10 (70%) | 9/11 (82%) | 10/12 (83%) | 13/14 (93%) | 16/20 (80%) |
| CRVS | 59/65 (91%) | 15/16 (94%) | 32/36 (89%) | 12/13 (92%) | 10/11 (91%) | 12/15 (80%) | 14/15 (93%) | 22/23 (96%) |
| Wastewater Surveillance | 47/60 (78%) | 11/15 (73%) | 26/34 (76%) | 10/11 (91%) | 7/10 (70%) | 7/13 (54%) | 12/15 (80%) | 20/21 (95%) |
| EBS: Human Health | 55/60 (92%) | 13/14 (93%) | 33/35 (94%) | 9/11 (82%) | 8/9 (89%) | 12/13 (92%) | 12/15 (80%) | 22/22 (100%) |
| EBS: Animal Health | 56/59 (95%) | 16/16 (100%) | 30/32 (94%) | 10/11 (91%) | 9/10 (90%) | 10/12 (83%) | 15/15 (100%) | 21/21 (100%) |
| EBS: Environmental Hazards | 52/60 (87%) | 14/16 (88%) | 29/33 (88%) | 9/11 (82%) | 7/10 (70%) | 10/13 (77%) | 13/14 (93%) | 21/22 (95%) |
| EBS: Biological hazards / biosecurity | 53/61 (87%) | 14/16 (88%) | 28/33 (85%) | 11/12 (92%) | 7/10 (70%) | 12/14 (86%) | 12/14 (86%) | 21/22 (95%) |
| EBS: Food & water security | 56/62 (90%) | 13/14 (93%) | 32/35 (91%) | 11/13 (85%) | 8/8 (100%) | 10/15 (67%) | 15/15 (100%) | 22/23 (96%) |

**Supplementary Table 2. Sectoral involvement in surveillance systems**

| **Sectors involved** | **Overall** | **IDS Status** | | | **WB Income Group** | | | |
| --- | --- | --- | --- | --- | --- | --- | --- | --- |
| **n/N (%)** |  | **Developed IDS** | **Partial IDS** | **No IDS** | **Low** | **Lower-middle** | **Upper-middle** | **High** |
| Public Sector Providers | 63/65 (97%) | 16/16 (100%) | 35/36 (97%) | 12/13 (92%) | 11/11 (100%) | 13/15 (87%) | 15/15 (100%) | 23/23 (100%) |
| Private Sector Providers | 52/65 (80%) | 13/16 (81%) | 31/36 (86%) | 8/13 (62%) | 8/11 (73%) | 9/15 (60%) | 14/15 (93%) | 20/23 (87%) |
| Public Health Sector | 63/65 (97%) | 16/16 (100%) | 35/36 (97%) | 12/13 (92%) | 10/11 (91%) | 14/15 (93%) | 15/15 (100%) | 23/23 (100%) |
| Animal Health Sector | 55/65 (85%) | 15/16 (94%) | 30/36 (83%) | 10/13 (77%) | 10/11 (91%) | 10/15 (67%) | 14/15 (93%) | 20/23 (87%) |
| Agricultural Sector | 35/65 (54%) | 12/16 (75%) | 17/36 (47%) | 6/13 (46%) | 8/11 (73%) | 6/15 (40%) | 7/15 (47%) | 13/23 (57%) |
| Environmental Sector | 46/65 (71%) | 14/16 (88%) | 25/36 (69%) | 7/13 (54%) | 7/11 (64%) | 7/15 (47%) | 13/15 (87%) | 18/23 (78%) |
| Laboratories | 64/65 (98%) | 16/16 (100%) | 36/36 (100%) | 12/13 (92%) | 11/11 (100%) | 14/15 (93%) | 15/15 (100%) | 23/23 (100%) |
| Private Sector | 39/65 (60%) | 11/16 (69%) | 24/36 (67%) | 4/13 (31%) | 3/11 (27%) | 8/15 (53%) | 12/15 (80%) | 15/23 (65%) |
| Communities | 31/65 (48%) | 12/16 (75%) | 17/36 (47%) | 2/13 (15%) | 11/11 (100%) | 8/15 (53%) | 6/15 (40%) | 6/23 (26%) |
| Pharmaceutical Sector | 28/65 (43%) | 8/16 (50%) | 17/36 (47%) | 3/13 (23%) | 3/11 (27%) | 4/15 (27%) | 8/15 (53%) | 12/23 (52%) |
| Occupational Health | 34/65 (52%) | 8/16 (50%) | 19/36 (53%) | 7/13 (54%) | 5/11 (45%) | 7/15 (47%) | 10/15 (67%) | 12/23 (52%) |
| Chemical and Poison Sector | 25/65 (38%) | 7/16 (44%) | 13/36 (36%) | 5/13 (38%) | 3/11 (27%) | 7/15 (47%) | 5/15 (33%) | 10/23 (43%) |
| Disaster Management | 38/65 (58%) | 8/16 (50%) | 26/36 (72%) | 4/13 (31%) | 4/11 (36%) | 7/15 (47%) | 11/15 (73%) | 15/23 (65%) |
| Food Safety | 46/65 (71%) | 10/16 (62%) | 29/36 (81%) | 7/13 (54%) | 5/11 (45%) | 8/15 (53%) | 12/15 (80%) | 20/23 (87%) |
| Biosafety and Biosecurity | 38/65 (58%) | 7/16 (44%) | 25/36 (69%) | 6/13 (46%) | 6/11 (55%) | 7/15 (47%) | 9/15 (60%) | 15/23 (65%) |

**Supplementary Table 3. Data collection mechanism for indicator-based surveillance programs**

| **Data collection mechanism** | **Overall** | **IDS Status** | | | **WB Income Group** | | | |
| --- | --- | --- | --- | --- | --- | --- | --- | --- |
| **n/N, %** |  | **Developed IDS** | **Partial IDS** | **No IDS** | **Low** | **Lower-middle** | **Upper-middle** | **High** |
| Notifiable disease surveillance |  |  |  |  |  |  |  |  |
| Digital | 19/62 (31%) | 6/16 (38%) | 11/35 (31%) | 2/11 (18%) | 2/11 (18%) | 3/12 (25%) | 4/15 (27%) | 9/23 (39%) |
| Paper | 3/62 (4.8%) | 0/16 (0%) | 2/35 (5.7%) | 1/11 (9.1%) | 1/11 (9.1%) | 0/12 (0%) | 1/15 (6.7%) | 1/23 (4.3%) |
| Hybrid | 40/62 (65%) | 10/16 (62%) | 22/35 (63%) | 8/11 (73%) | 8/11 (73%) | 9/12 (75%) | 10/15 (67%) | 13/23 (57%) |
| Mandatory notification |  |  |  |  |  |  |  |  |
| Digital | 17/60 (28%) | 6/16 (38%) | 11/34 (32%) | 0/10 (0%) | 2/10 (20%) | 1/11 (9.1%) | 4/15 (27%) | 9/23 (39%) |
| Paper | 6/60 (10%) | 0/16 (0%) | 4/34 (12%) | 2/10 (20%) | 2/10 (20%) | 1/11 (9.1%) | 1/15 (6.7%) | 2/23 (8.7%) |
| Hybrid | 37/60 (62%) | 10/16 (62%) | 19/34 (56%) | 8/10 (80%) | 6/10 (60%) | 9/11 (82%) | 10/15 (67%) | 12/23 (52%) |
| Sentinel surveillance |  |  |  |  |  |  |  |  |
| Digital | 26/60 (43%) | 6/15 (40%) | 18/33 (55%) | 2/12 (17%) | 2/10 (20%) | 3/13 (23%) | 5/13 (38%) | 15/23 (65%) |
| Paper | 5/60 (8.3%) | 1/15 (6.7%) | 1/33 (3.0%) | 3/12 (25%) | 1/10 (10%) | 2/13 (15%) | 0/13 (0%) | 2/23 (8.7%) |
| Hybrid | 29/60 (48%) | 8/15 (53%) | 14/33 (42%) | 7/12 (58%) | 7/10 (70%) | 8/13 (62%) | 8/13 (62%) | 6/23 (26%) |
| Syndromic surveillance |  |  |  |  |  |  |  |  |
| Digital | 26/56 (46%) | 6/13 (46%) | 17/32 (53%) | 3/11 (27%) | 2/10 (20%) | 3/12 (25%) | 4/13 (31%) | 16/20 (80%) |
| Paper | 2/56 (3.6%) | 1/13 (7.7%) | 0/32 (0%) | 1/11 (9.1%) | 1/10 (10%) | 1/12 (8.3%) | 0/13 (0%) | 0/20 (0%) |
| Hybrid | 28/56 (50%) | 6/13 (46%) | 15/32 (47%) | 7/11 (64%) | 7/10 (70%) | 8/12 (67%) | 9/13 (69%) | 4/20 (20%) |
| Registers (Routine health indicators) |  |  |  |  |  |  |  |  |
| Digital | 15/57 (26%) | 4/15 (27%) | 11/32 (34%) | 0/10 (0%) | 1/11 (9.1%) | 0/11 (0%) | 4/15 (27%) | 10/20 (50%) |
| Paper | 9/57 (16%) | 2/15 (13%) | 4/32 (12%) | 3/10 (30%) | 4/11 (36%) | 4/11 (36%) | 1/15 (6.7%) | 0/20 (0%) |
| Hybrid | 33/57 (58%) | 9/15 (60%) | 17/32 (53%) | 7/10 (70%) | 6/11 (55%) | 7/11 (64%) | 10/15 (67%) | 10/20 (50%) |
| Mortality data |  |  |  |  |  |  |  |  |
| Digital | 18/57 (32%) | 4/14 (29%) | 13/33 (39%) | 1/10 (10%) | 1/10 (10%) | 1/9 (11%) | 5/15 (33%) | 10/22 (45%) |
| Paper | 7/57 (12%) | 1/14 (7.1%) | 4/33 (12%) | 2/10 (20%) | 2/10 (20%) | 2/9 (22%) | 2/15 (13%) | 1/22 (4.5%) |
| Hybrid | 32/57 (56%) | 9/14 (64%) | 16/33 (48%) | 7/10 (70%) | 7/10 (70%) | 6/9 (67%) | 8/15 (53%) | 11/22 (50%) |
| Laboratory data |  |  |  |  |  |  |  |  |
| Digital | 28/62 (45%) | 5/16 (31%) | 19/35 (54%) | 4/11 (36%) | 0/11 (0%) | 2/12 (17%) | 8/15 (53%) | 17/23 (74%) |
| Paper | 5/62 (8.1%) | 2/16 (12%) | 1/35 (2.9%) | 2/11 (18%) | 3/11 (27%) | 2/12 (17%) | 0/15 (0%) | 0/23 (0%) |
| Hybrid | 29/62 (47%) | 9/16 (56%) | 15/35 (43%) | 5/11 (45%) | 8/11 (73%) | 8/12 (67%) | 7/15 (47%) | 6/23 (26%) |
| Surveys and research |  |  |  |  |  |  |  |  |
| Digital | 9/55 (16%) | 2/14 (14%) | 7/31 (23%) | 0/10 (0%) | 0/9 (0%) | 0/11 (0%) | 3/12 (25%) | 6/22 (27%) |
| Paper | 4/55 (7.3%) | 0/14 (0%) | 3/31 (9.7%) | 1/10 (10%) | 1/9 (11%) | 2/11 (18%) | 0/12 (0%) | 0/22 (0%) |
| Hybrid | 42/55 (76%) | 12/14 (86%) | 21/31 (68%) | 9/10 (90%) | 8/9 (89%) | 9/11 (82%) | 9/12 (75%) | 16/22 (73%) |
| Hospital discharge data |  |  |  |  |  |  |  |  |
| Digital | 24/57 (42%) | 4/13 (31%) | 15/34 (44%) | 5/10 (50%) | 1/10 (10%) | 0/11 (0%) | 4/14 (29%) | 18/21 (86%) |
| Paper | 7/57 (12%) | 4/13 (31%) | 2/34 (5.9%) | 1/10 (10%) | 3/10 (30%) | 4/11 (36%) | 0/14 (0%) | 0/21 (0%) |
| Hybrid | 26/57 (46%) | 5/13 (38%) | 17/34 (50%) | 4/10 (40%) | 6/10 (60%) | 7/11 (64%) | 10/14 (71%) | 3/21 (14%) |
| Genomic surveillance |  |  |  |  |  |  |  |  |
| Digital | 29/46 (63%) | 6/10 (60%) | 19/28 (68%) | 4/8 (50%) | 3/6 (50%) | 3/9 (33%) | 6/11 (55%) | 17/20 (85%) |
| Paper | 2/46 (4.3%) | 0/10 (0%) | 2/28 (7.1%) | 0/8 (0%) | 0/6 (0%) | 1/9 (11%) | 1/11 (9.1%) | 0/20 (0%) |
| Hybrid | 15/46 (33%) | 4/10 (40%) | 7/28 (25%) | 4/8 (50%) | 3/6 (50%) | 5/9 (56%) | 4/11 (36%) | 3/20 (15%) |
| Behavioural surveillance |  |  |  |  |  |  |  |  |
| Digital | 10/28 (36%) | 2/5 (40%) | 6/19 (32%) | 2/4 (50%) | 0/1 (0%) | 0/6 (0%) | 2/6 (33%) | 8/15 (53%) |
| Paper | 4/28 (14%) | 0/5 (0%) | 3/19 (16%) | 1/4 (25%) | 0/1 (0%) | 2/6 (33%) | 1/6 (17%) | 1/15 (6.7%) |
| Hybrid | 14/28 (50%) | 3/5 (60%) | 10/19 (53%) | 1/4 (25%) | 1/1 (100%) | 4/6 (67%) | 3/6 (50%) | 6/15 (40%) |
| Vaccine coverage |  |  |  |  |  |  |  |  |
| Digital | 23/60 (38%) | 4/14 (29%) | 15/35 (43%) | 4/11 (36%) | 1/11 (9.1%) | 1/10 (10%) | 7/15 (47%) | 13/23 (57%) |
| Paper | 2/60 (3.3%) | 1/14 (7.1%) | 0/35 (0%) | 1/11 (9.1%) | 2/11 (18%) | 0/10 (0%) | 0/15 (0%) | 0/23 (0%) |
| Hybrid | 35/60 (58%) | 9/14 (64%) | 20/35 (57%) | 6/11 (55%) | 8/11 (73%) | 9/10 (90%) | 8/15 (53%) | 10/23 (43%) |

**Supplementary Table 4. Data transfer mechanism for integrating lab data into IDS system**

| **Data transfer mechanism** | **Overall** | **IDS Status** | | | **WB Income Group** | | | |
| --- | --- | --- | --- | --- | --- | --- | --- | --- |
| **n/N (%)** |  | **Developed IDS** | **Partial IDS** | **No IDS** | **Low** | **Lower-middle** | **Upper-middle** | **High** |
| IT / Electronic Systems | 54/61 (89%) | 12/15 (80%) | 30/33 (91%) | 12/13 (92%) | 9/11 (82%) | 13/15 (87%) | 12/15 (80%) | 19/19 (100%) |
| Phone / Text / Fax | 20/61 (33%) | 3/15 (20%) | 13/33 (39%) | 4/13 (31%) | 3/11 (27%) | 8/15 (53%) | 4/15 (27%) | 5/19 (26%) |
| Manually | 22/61 (36%) | 4/15 (27%) | 12/33 (36%) | 6/13 (46%) | 3/11 (27%) | 9/15 (60%) | 5/15 (33%) | 5/19 (26%) |

**Supplementary Table 5. Establishment of privacy protection for public health surveillance systems**

| **Level of protection** | **Overall** | **IDS Status** | | | **WB Income Group** | | | |
| --- | --- | --- | --- | --- | --- | --- | --- | --- |
| **n/N (%)** |  | **Developed IDS** | **Partial IDS** | **No IDS** | **Low** | **Lower-middle** | **Upper-middle** | **High** |
| Well established | 31/63 (49%) | 9/16 (56%) | 18/35 (51%) | 4/12 (33%) | 4/11 (36%) | 4/14 (29%) | 7/15 (47%) | 15/22 (68%) |
| Partial or in development | 26/63 (41%) | 6/16 (38%) | 15/35 (43%) | 5/12 (42%) | 5/11 (45%) | 7/14 (50%) | 8/15 (53%) | 6/22 (27%) |
| None | 6/63 (9.5%) | 1/16 (6.2%) | 2/35 (5.7%) | 3/12 (25%) | 2/11 (18%) | 3/14 (21%) | 0/15 (0%) | 1/22 (4.5%) |

**Supplementary Table 6. Workforce capacity for event-based and indicator-based surveillance systems**

| **EBS Workforce Capacity level** | **Overall** | **IDS Status** | | | **WB Income Group** | | | |
| --- | --- | --- | --- | --- | --- | --- | --- | --- |
| **n/N (%)** |  | **Developed IDS** | **Partial IDS** | **No IDS** | **Low** | **Lower-middle** | **Upper-middle** | **High** |
| Weak | 15/63 (24%) | 3/15 (20%) | 8/35 (23%) | 4/13 (31%) | 5/11 (45%) | 5/14 (36%) | 2/15 (13%) | 3/22 (14%) |
| Average | 44/63 (70%) | 11/15 (73%) | 25/35 (71%) | 8/13 (62%) | 6/11 (55%) | 9/14 (64%) | 12/15 (80%) | 16/22 (73%) |
| Strong | 4/63 (6.3%) | 1/15 (6.7%) | 2/35 (5.7%) | 1/13 (7.7%) | 0/11 (0%) | 0/14 (0%) | 1/15 (6.7%) | 3/22 (14%) |
|  |  |  |  |  |  |  |  |  |
| **IBS Workforce Capacity level** | **Overall** | **IDS Status** | | | **WB Income Group** | | | |
| **n/N (%)** |  | **Developed IDS** | **Partial IDS** | **No IDS** | **Low** | **Lower-middle** | **Upper-middle** | **High** |
| Weak | 5/60 (8.3%) | 1/14 (7.1%) | 2/34 (5.9%) | 2/12 (17%) | 1/10 (10%) | 1/14 (7.1%) | 2/15 (13%) | 1/20 (5.0%) |
| Average | 50/60 (83%) | 13/14 (93%) | 28/34 (82%) | 9/12 (75%) | 9/10 (90%) | 13/14 (93%) | 12/15 (80%) | 15/20 (75%) |
| Strong | 5/60 (8.3%) | 0/14 (0%) | 4/34 (12%) | 1/12 (8.3%) | 0/10 (0%) | 0/14 (0%) | 1/15 (6.7%) | 4/20 (20%) |

**Supplementary Table 7. Workforce capacity gaps**

| **Workforce Capacity Gaps** | **Overall** | **IDS Status** | | | **WB Income Group** | | | |
| --- | --- | --- | --- | --- | --- | --- | --- | --- |
| **n/N (%)** |  | **Developed IDS** | **Partial IDS** | **No IDS** | **Low** | **Lower-middle** | **Upper-middle** | **High** |
| Laboratory | 33/65 (51%) | 12/16 (75%) | 17/36 (47%) | 4/13 (31%) | 7/11 (64%) | 9/15 (60%) | 6/15 (40%) | 10/23 (43%) |
| Epidemiology | 39/65 (60%) | 11/16 (69%) | 20/36 (56%) | 8/13 (62%) | 8/11 (73%) | 9/15 (60%) | 7/15 (47%) | 14/23 (61%) |
| Admin / Data Entry | 35/65 (54%) | 8/16 (50%) | 19/36 (53%) | 8/13 (62%) | 6/11 (55%) | 11/15 (73%) | 8/15 (53%) | 9/23 (39%) |
| Data Science & Analytics | 56/65 (86%) | 14/16 (88%) | 33/36 (92%) | 9/13 (69%) | 11/11 (100%) | 12/15 (80%) | 13/15 (87%) | 19/23 (83%) |
| Public Health Generalist | 20/65 (31%) | 4/16 (25%) | 11/36 (31%) | 5/13 (38%) | 0/11 (0%) | 6/15 (40%) | 3/15 (20%) | 10/23 (43%) |
| IT | 49/65 (75%) | 10/16 (62%) | 27/36 (75%) | 12/13 (92%) | 9/11 (82%) | 12/15 (80%) | 9/15 (60%) | 18/23 (78%) |
| None | 1/65 (1.5%) | 0/16 (0%) | 1/36 (2.8%) | 0/13 (0%) | 0/11 (0%) | 0/15 (0%) | 1/15 (6.7%) | 0/23 (0%) |

**Supplementary Table 8. Workforce development initiatives leading organisations**

| **Workforce development initiatives led by** | **Overall** | **IDS Status** | | | **WB Income Group** | | | |
| --- | --- | --- | --- | --- | --- | --- | --- | --- |
| **n/N (%)** |  | **Developed IDS** | **Partial IDS** | **No IDS** | **Low** | **Lower-middle** | **Upper-middle** | **High** |
| NPHI | 38/65 (58%) | 8/16 (50%) | 23/36 (64%) | 7/13 (54%) | 9/11 (82%) | 7/15 (47%) | 11/15 (73%) | 11/23 (48%) |
| MOH | 39/65 (60%) | 6/16 (38%) | 24/36 (67%) | 8/13 (62%) | 9/11 (82%) | 10/15 (67%) | 9/15 (60%) | 9/23 (39%) |
| In-country academic institution | 20/65 (31%) | 5/16 (31%) | 12/36 (33%) | 2/13 (15%) | 7/11 (64%) | 4/15 (27%) | 2/15 (13%) | 6/23 (26%) |
| Private industry | 1/65 (1.5%) | 0/16 (0%) | 1/36 (2.8%) | 0/13 (0%) | 0/11 (0%) | 1/15 (6.7%) | 0/15 (0%) | 0/23 (0%) |
| Externally supported/funded | 19/65 (29%) | 3/16 (19%) | 12/36 (33%) | 4/13 (31%) | 5/11 (45%) | 5/15 (33%) | 3/15 (20%) | 5/23 (22%) |
| None | 4/65 (6.2%) | 2/16 (12%) | 2/36 (5.6%) | 0/13 (0%) | 1/11 (9.1%) | 0/15 (0%) | 1/15 (6.7%) | 2/23 (8.7%) |
| Other | 3/65 (4.6%) | 2/16 (12%) | 1/36 (2.8%) | 0/13 (0%) | 0/11 (0%) | 0/15 (0%) | 1/15 (6.7%) | 2/23 (8.7%) |

**Supplementary Table 9. Legal mandate for notifiable disease/hazard reporting**

| **Legal mandate for notifiable disease / hazard reporting** | **Overall** | **IDS Status** | | | **WB Income Group** | | | |
| --- | --- | --- | --- | --- | --- | --- | --- | --- |
| **n/N (%)** |  | **Developed IDS** | **Partial IDS** | **No IDS** | **Low** | **Lower-middle** | **Upper-middle** | **High** |
| MOH | 33/64 (52%) | 5/16 (31%) | 19/35 (54%) | 9/13 (69%) | 7/11 (64%) | 9/14 (64%) | 8/15 (53%) | 9/23 (39%) |
| NPHI | 11/64 (17%) | 2/16 (12%) | 7/35 (20%) | 2/13 (15%) | 0/11 (0%) | 1/14 (7.1%) | 2/15 (13%) | 8/23 (35%) |
| Both | 12/64 (19%) | 6/16 (38%) | 5/35 (14%) | 1/13 (7.7%) | 4/11 (36%) | 1/14 (7.1%) | 4/15 (27%) | 3/23 (13%) |
| None | 2/64 (3.1%) | 1/16 (6.2%) | 0/35 (0%) | 1/13 (7.7%) | 0/11 (0%) | 2/14 (14%) | 0/15 (0%) | 0/23 (0%) |
| Other | 6/64 (9.4%) | 2/16 (12%) | 4/35 (11%) | 0/13 (0%) | 0/11 (0%) | 1/14 (7.1%) | 1/15 (6.7%) | 3/23 (13%) |

| Other: |  |  |  |  |  |  |  |  |
| --- | --- | --- | --- | --- | --- | --- | --- | --- |
| Authority, but lack of enforcement (2) | | | |  |  |  |  |  |
| Policy, but not law (2) | | |  |  |  |  |  |  |
| Other organizations including: | | | |  |  |  |  |  |
| - Ministry of Defense | | | |  |  |  |  |  |
| - Ministry of Interior | | | |  |  |  |  |  |
| - Regional public health authority | | | | |  |  |  |  |
| - National patient safety authority | | | | |  |  |  |  |
| - Food / Consumer Safety | | | |  |  |  |  |  |
| - National emergency management | | | | |  |  |  |  |
| - WHO | | |  |  |  |  |  |  |

**Supplementary Table 10. Notifiable conditions**

| **Notifiable conditions** | **Overall** | **IDS Status** | | | **WB Income Group** | | | |
| --- | --- | --- | --- | --- | --- | --- | --- | --- |
| **n/N, %** |  | **Developed IDS** | **Partial IDS** | **No IDS** | **Low** | **Lower-middle** | **Upper-middle** | **High** |
| Communicable | 61/62 (98%) | 14/15 (93%) | 35/35 (100%) | 12/12 (100%) | 10/11 (91%) | 12/12 (100%) | 15/15 (100%) | 23/23 (100%) |
| Non-communicable | 28/62 (45%) | 8/15 (53%) | 15/35 (43%) | 5/12 (42%) | 6/11 (55%) | 4/12 (33%) | 9/15 (60%) | 8/23 (35%) |
| Causative agents | 34/62 (55%) | 10/15 (67%) | 21/35 (60%) | 3/12 (25%) | 5/11 (45%) | 4/12 (33%) | 7/15 (47%) | 17/23 (74%) |
| Chemical / radiation hazards | 39/62 (63%) | 9/15 (60%) | 24/35 (69%) | 6/12 (50%) | 6/11 (55%) | 7/12 (58%) | 11/15 (73%) | 14/23 (61%) |
| Environmental hazards | 33/62 (53%) | 10/15 (67%) | 20/35 (57%) | 3/12 (25%) | 6/11 (55%) | 5/12 (42%) | 10/15 (67%) | 11/23 (48%) |
| Disease in animals | 47/62 (76%) | 12/15 (80%) | 30/35 (86%) | 5/12 (42%) | 9/11 (82%) | 5/12 (42%) | 13/15 (87%) | 19/23 (83%) |

**Supplementary Table 11. Level of adherence to notifiable disease/hazard reporting mandate**

| **Level of adherence to reporting mandate** | **Overall** | **IDS Status** | | | **WB Income Group** | | | |
| --- | --- | --- | --- | --- | --- | --- | --- | --- |
| **n/N, %** |  | **Developed IDS** | **Partial IDS** | **No IDS** | **Low** | **Lower-middle** | **Upper-middle** | **High** |
| Poor adherence | 6/59 (10%) | 0/12 (0%) | 3/35 (8.6%) | 3/12 (25%) | 7/11 (64%) | 11/15 (73%) | 10/15 (67%) | 14/23 (61%) |
| Partial adherence | 30/59 (51%) | 7/12 (58%) | 17/35 (49%) | 6/12 (50%) | 4/11 (36%) | 3/15 (20%) | 2/15 (13%) | 3/23 (13%) |
| Good adherence | 23/59 (39%) | 5/12 (42%) | 15/35 (43%) | 3/12 (25%) | 0/11 (0%) | 1/15 (6.7%) | 3/15 (20%) | 6/23 (26%) |

**Supplementary Table 12. Lead agency for public health surveillance**

| **Lead Agency for Public Health Surveillance** | **Overall** | **IDS Status** | | | **WB Income Group** | | | | **WHO Region** | | | | | |
| --- | --- | --- | --- | --- | --- | --- | --- | --- | --- | --- | --- | --- | --- | --- |
| **n/N (%)** |  | **Developed IDS** | **Partial IDS** | **No IDS** | **Low** | **Lower-middle** | **Upper-middle** | **High** | **Africa** | **Americas** | **Eastern Mediterranean** | **Europe** | **Southeast Asia** | **Western Pacific** |
| NPHI | 21/65 (32%) | 6/16 (38%) | 13/36 (36%) | 2/13 (15%) | 2/11 (18%) | 4/15 (27%) | 4/15 (27%) | 11/23 (48%) | 3/17 (18%) | 1/15 (6.7%) | 2/5  (40%) | 14/22 (64%) | 0/2  (0%) | 1/4 (25%) |
| MOH | 29/65 (45%) | 7/16 (44%) | 14/36 (39%) | 8/13 (62%) | 7/11 (64%) | 9/15 (60%) | 6/15 (40%) | 7/23 (30%) | 10/17 (59%) | 10/15 (67%) | 3/5  (60%) | 3/22 (14%) | 1/2  (50%) | 2/4 (50%) |
| Joint NPHI/MOH | 7/65 (11%) | 0/16 (0%) | 5/36 (14%) | 2/13 (15%) | 2/11 (18%) | 1/15 (6.7%) | 3/15 (20%) | 1/23 (4.3%) | 2/17 (12%) | 1/15 (6.7%) | 0/5  (0%) | 3/22 (14%) | 1/2  (50%) | 0/4  (0%) |
| Other | 8/65 (12%) | 3/16 (19%) | 4/36 (11%) | 1/13 (7.7%) | 0/11 (0%) | 1/15 (6.7%) | 2/15 (13%) | 4/23 (17%) | 2/17 (12%) | 3/15 (20%) | 0/5  (0%) | 2/22 (9.1%) | 0/2  (0%) | 1/4 (25%) |

| Other includes: |
| --- |
| ∙       Regional NPHI (1) |
| ∙       Multiple organizations in addition to MOH and/or NPHI (5) |
| ∙       NPHI at a national level and other subnational institutions (2) |

**Supplementary Table 13. National Public Health Institution IDS responsibility**

| **NPHI IDS responsibility** | **Overall** | **IDS Status** | | **WB Income Group** | | | |
| --- | --- | --- | --- | --- | --- | --- | --- |
| **n/N (%)** |  | **Developed IDS** | **Partial IDS** | **Low** | **Lower-middle** | **Upper-middle** | **High** |
| Sole | 10/50 (20%) | 7/34 (21%) | 3/16 (19%) | 4/9 (44%) | 4/11 (36%) | 1/13 (8%) | 1/17 (6%) |
| Joint | 30/50 (60%) | 20/34 (59%) | 10/16 (63%) | 4/9 (44%) | 5/11 (45%) | 8/13 (62%) | 13/17 (76%) |
| Not responsible | 10/50 (20%) | 7/34 (21%) | 3/16 (19%) | 1/9 (11%) | 2/11 (18%) | 4/13 (31%) | 3/17 (18%) |

**Supplementary Table 14. Lead agency by function**

| **Lead agency by function** | **Overall** | | | | | **IDS Status** | | | | | | | | | |
| --- | --- | --- | --- | --- | --- | --- | --- | --- | --- | --- | --- | --- | --- | --- | --- |
| **n/N (%)** |  |  |  |  |  | **Developed IDS** | | | | | **Partial IDS** | | | | |
| **Functions** | **NPHI** | **MOH** | **COS** | **No lead** | **Other** | **NPHI** | **MOH** | **COS** | **No lead** | **Other** | **NPHI** | **MOH** | **COS** | **No lead** | **Other** |
| Detection | 33/49 (68%) | 22/49 (45%) | 1/49 (2%) | 0/49 (0%) | 12/49 (24%) | 22/33 (67%) | 14/33 (42%) | 0/33 (0%) | 0/33 (0%) | 9/33 (27%) | 11/16 (69%) | 8/16 (50%) | 1/16 (6%) | 0/16 (0%) | 3/16 (19%) |
| Reporting | 31/49 (63%) | 20/49 (41%) | 2/49 (5%) | 0/49 (0%) | 9/49 (19%) | 20/33 (61%) | 13/33 (40%) | 1/33 (4%) | 0/33 (0%) | 6/33 (18%) | 11/16 (69%) | 7/16 (44%) | 1/16 (7%) | 0/16 (0%) | 3/16 (19%) |
| Analysis | 38/49 (78%) | 18/49 (37%) | 8/49 (16%) | 0/49 (0%) | 5/49 (10%) | 25/33 (76%) | 12/33 (37%) | 6/33 (18%) | 0/33 (0%) | 2/33 (5%) | 13/16 (81%) | 6/16 (38%) | 2/16 (12%) | 0/16 (0%) | 3/16 (20%) |
| Investigation | 36/48 (75%) | 21/48 (44%) | 0/48 (0%) | 0/48 (0%) | 9/48 (18%) | 25/33 (75%) | 13/33 (39%) | 0/33 (0%) | 0/33 (0%) | 8/33 (24%) | 11/15 (73%) | 8/15 (53%) | 0/15 (0%) | 0/15 (0%) | 1/15 (7%) |
| Response | 28/48 (59%) | 28/48 (59%) | 0/48 (0%) | 0/48 (0%) | 12/48 (25%) | 21/32 (66%) | 19/32 (60%) | 0/32 (0%) | 0/32 (0%) | 8/32 (26%) | 7/16 (44%) | 9/16 (56%) | 0/16 (0%) | 0/16 (0%) | 4/16 (25%) |
| Feedback | 29/45 (65%) | 26/45 (57%) | 0/45 (0%) | 1/45 (2%) | 9/45 (20%) | 21/31 (68%) | 18/31 (58%) | 0/31 (0%) | 1/31 (3%) | 7/31 (23%) | 8/14 (57%) | 8/14 (57%) | 0/14 (0%) | 0/14 (0%) | 2/14 (14%) |
| Evaluation | 33/48 (69%) | 26/48 (54%) | 1/48 (2%) | 1/48 (2%) | 9/48 (18%) | 22/32 (69%) | 19/32 (60%) | 1/32 (3%) | 1/32 (3%) | 6/32 (18%) | 11/16 (68%) | 7/16 (43%) | 0/16 (0%) | 0/16 (0%) | 3/16 (18%) |
| Preparedness | 33/47 (70%) | 31/47 (66%) | 1/47 (2%) | 1/47 (2%) | 10/47 (21%) | 23/32 (73%) | 22/32 (69%) | 1/32 (3%) | 1/32 (3%) | 7/32 (22%) | 10/15 (66%) | 9/15 (60%) | 0/15 (0%) | 0/15 (0%) | 3/15 (21%) |

NPHI: National Public Health Institute

MOH: Ministry of Health

COS: Center of Statistics

**Supplementary Table 15. Level of surveillance system integration**

| **Level of Surveillance System Integration** | **Overall** | **WB Income Group** | | | | **WHO Region** | | | | | |
| --- | --- | --- | --- | --- | --- | --- | --- | --- | --- | --- | --- |
| **n/N (%)** |  | **Low** | **Lower-middle** | **Upper-middle** | **High** | **Africa** | **Americas** | **Eastern Mediterranean** | **Europe** | **Southeast Asia** | **Western Pacific** |
| Developed IDS | 16/65 (25%) | 5/11 (45%) | 5/15 (33%) | 2/15 (13%) | 4/23 (17%) | 10/17 (59%) | 2/14 (14%) | 0/5  (0%) | 4/22 (18%) | 0/2  (0%) | 0/4  (0%) |
| Partial IDS | 36/65 (55%) | 4/11 (36%) | 6/15 (40%) | 11/15 (73%) | 14/23 (61%) | 5/17 (29%) | 8/14 (57%) | 2/5  (40%) | 17/22 (77%) | 1/2  (50%) | 2/4  (50%) |
| No IDS | 13/65 (20%) | 2/11 (18%) | 4/15 (27%) | 2/15 (13%) | 5/23 (22%) | 2/17 (12%) | 4/14 (29%) | 3/5  (60%) | 1/22 (5%) | 1/2  (50%) | 2/4  (50%) |

**Supplementary Table 16. Data collected through surveillance systems**

| **Data collected through surveillance system** | **Overall** | **IDS Status** | | | **WB Income Group** | | | |
| --- | --- | --- | --- | --- | --- | --- | --- | --- |
| **n/N (%)** |  | **Developed IDS** | **Partial IDS** | **No IDS** | **Low** | **Lower-middle** | **Upper-middle** | **High** |
| Notifiable Diseases | 64/64 (100%) | 16/16 (100%) | 36/36 (100%) | 12/12 (100%) | 11/11 (100%) | 14/14 (100%) | 15/15 (100%) | 23/23 (100%) |
| Syndromic Surveillance | 62/65 (95%) | 16/16 (100%) | 34/36 (94%) | 12/13 (92%) | 11/11 (100%) | 15/15 (100%) | 14/15 (93%) | 21/23 (91%) |
| Sentinel Surveillance | 63/63 (100%) | 16/16 (100%) | 35/35 (100%) | 12/12 (100%) | 11/11 (100%) | 13/13 (100%) | 15/15 (100%) | 23/23 (100%) |
| Lab-based Surveillance | 63/64 (98%) | 16/16 (100%) | 35/35 (100%) | 12/13 (92%) | 11/11 (100%) | 13/14 (93%) | 15/15 (100%) | 23/23 (100%) |
| Hospital-based Surveillance | 62/64 (97%) | 15/16 (94%) | 34/35 (97%) | 13/13 (100%) | 11/11 (100%) | 14/14 (100%) | 15/15 (100%) | 22/23 (96%) |
| Other Facility-based Surveillance | 59/60 (98%) | 16/16 (100%) | 31/32 (97%) | 12/12 (100%) | 11/11 (100%) | 13/13 (100%) | 14/14 (100%) | 20/21 (95%) |
| Case-based Surveillance | 61/62 (98%) | 16/16 (100%) | 33/34 (97%) | 12/12 (100%) | 11/11 (100%) | 13/13 (100%) | 14/14 (100%) | 23/23 (100%) |
| Disease-specific Surveillance | 64/64 (100%) | 16/16 (100%) | 36/36 (100%) | 12/12 (100%) | 11/11 (100%) | 14/14 (100%) | 15/15 (100%) | 23/23 (100%) |
| Community-based Surveillance | 46/60 (77%) | 15/16 (94%) | 24/32 (75%) | 7/12 (58%) | 11/11 (100%) | 10/13 (77%) | 10/15 (67%) | 14/20 (70%) |
| Mortality Surveillance | 60/65 (92%) | 14/16 (88%) | 35/36 (97%) | 11/13 (85%) | 9/11 (82%) | 12/15 (80%) | 15/15 (100%) | 23/23 (100%) |
| Genomic Surveillance | 57/64 (89%) | 16/16 (100%) | 30/35 (86%) | 11/13 (85%) | 9/11 (82%) | 13/15 (87%) | 11/14 (79%) | 23/23 (100%) |
| Behavioral Surveillance | 35/56 (62%) | 9/13 (69%) | 21/33 (64%) | 5/10 (50%) | 5/9 (56%) | 6/11 (55%) | 7/14 (50%) | 17/22 (77%) |
| Vaccination Coverage | 64/64 (100%) | 16/16 (100%) | 36/36 (100%) | 12/12 (100%) | 11/11 (100%) | 14/14 (100%) | 15/15 (100%) | 23/23 (100%) |
| Vaccine Effectiveness | 59/63 (94%) | 15/16 (94%) | 33/35 (94%) | 11/12 (92%) | 10/11 (91%) | 12/14 (86%) | 13/14 (93%) | 23/23 (100%) |
| Survey and Research | 49/58 (84%) | 15/16 (94%) | 27/32 (84%) | 7/10 (70%) | 9/11 (82%) | 10/12 (83%) | 13/14 (93%) | 16/20 (80%) |
| CRVS | 59/65 (91%) | 15/16 (94%) | 32/36 (89%) | 12/13 (92%) | 10/11 (91%) | 12/15 (80%) | 14/15 (93%) | 22/23 (96%) |
| Wastewater Surveillance | 47/60 (78%) | 11/15 (73%) | 26/34 (76%) | 10/11 (91%) | 7/10 (70%) | 7/13 (54%) | 12/15 (80%) | 20/21 (95%) |
| EBS: Human Health | 55/60 (92%) | 13/14 (93%) | 33/35 (94%) | 9/11 (82%) | 8/9 (89%) | 12/13 (92%) | 12/15 (80%) | 22/22 (100%) |
| EBS: Animal Health | 56/59 (95%) | 16/16 (100%) | 30/32 (94%) | 10/11 (91%) | 9/10 (90%) | 10/12 (83%) | 15/15 (100%) | 21/21 (100%) |
| EBS: Environmental Hazards | 52/60 (87%) | 14/16 (88%) | 29/33 (88%) | 9/11 (82%) | 7/10 (70%) | 10/13 (77%) | 13/14 (93%) | 21/22 (95%) |
| EBS: Biological hazards / biosecurity | 53/61 (87%) | 14/16 (88%) | 28/33 (85%) | 11/12 (92%) | 7/10 (70%) | 12/14 (86%) | 12/14 (86%) | 21/22 (95%) |
| EBS: Food & water security | 56/62 (90%) | 13/14 (93%) | 32/35 (91%) | 11/13 (85%) | 8/8 (100%) | 10/15 (67%) | 15/15 (100%) | 22/23 (96%) |

**Supplementary Table 17. Data collected through surveillance systems are accessible**

| **Data collected through system are accessible** | **Overall** | **IDS Status** | | | **WB Income Group** | | | |
| --- | --- | --- | --- | --- | --- | --- | --- | --- |
| **n/N (%)** |  | **Developed IDS** | **Partial IDS** | **No IDS** | **Low** | **Lower-middle** | **Upper-middle** | **High** |
| Notifiable Diseases | 63/64 (98%) | 16/16 (100%) | 35/36 (97%) | 12/12 (100%) | 11/11 (100%) | 14/14 (100%) | 15/15 (100%) | 22/23 (96%) |
| Syndromic Surveillance | 57/62 (92%) | 15/16 (94%) | 31/34 (91%) | 11/12 (92%) | 10/11 (91%) | 13/15 (87%) | 14/14 (100%) | 19/21 (90%) |
| Sentinel Surveillance | 62/63 (98%) | 15/16 (94%) | 35/35 (100%) | 12/12 (100%) | 11/11 (100%) | 13/13 (100%) | 15/15 (100%) | 22/23 (96%) |
| Lab-based Surveillance | 61/63 (97%) | 16/16 (100%) | 34/35 (97%) | 11/12 (92%) | 11/11 (100%) | 12/13 (92%) | 15/15 (100%) | 22/23 (96%) |
| Hospital-based Surveillance | 50/62 (81%) | 14/15 (93%) | 26/34 (76%) | 10/13 (77%) | 10/11 (91%) | 10/14 (71%) | 12/15 (80%) | 18/22 (82%) |
| Other Facility-based Surveillance | 46/59 (78%) | 13/16 (81%) | 24/31 (77%) | 9/12 (75%) | 8/11 (73%) | 10/13 (77%) | 11/14 (79%) | 16/20 (80%) |
| Case-based Surveillance | 59/61 (97%) | 16/16 (100%) | 32/33 (97%) | 11/12 (92%) | 11/11 (100%) | 12/13 (92%) | 13/14 (93%) | 23/23 (100%) |
| Disease-specific Surveillance | 61/64 (95%) | 16/16 (100%) | 34/36 (94%) | 11/12 (92%) | 11/11 (100%) | 12/14 (86%) | 14/15 (93%) | 23/23 (100%) |
| Community-based Surveillance | 40/46 (87%) | 13/15 (87%) | 20/24 (83%) | 7/7 (100%) | 10/11 (91%) | 9/10 (90%) | 8/10 (80%) | 12/14 (86%) |
| Mortality Surveillance | 49/60 (82%) | 12/14 (86%) | 28/35 (80%) | 9/11 (82%) | 7/9 (78%) | 8/12 (67%) | 13/15 (87%) | 20/23 (87%) |
| Genomic Surveillance | 45/57 (79%) | 12/16 (75%) | 24/30 (80%) | 9/11 (82%) | 7/9 (78%) | 10/13 (77%) | 8/11 (73%) | 19/23 (83%) |
| Behavioral Surveillance | 23/35 (66%) | 6/9 (67%) | 13/21 (62%) | 4/5 (80%) | 1/5 (20%) | 4/6 (67%) | 4/7 (57%) | 14/17 (82%) |
| Vaccination Coverage | 58/64 (91%) | 15/16 (94%) | 32/36 (89%) | 11/12 (92%) | 11/11 (100%) | 14/14 (100%) | 14/15 (93%) | 19/23 (83%) |
| Vaccine Effectiveness | 48/59 (81%) | 14/15 (93%) | 26/33 (79%) | 8/11 (73%) | 8/10 (80%) | 11/12 (92%) | 11/13 (85%) | 18/23 (78%) |
| Survey and Research | 37/49 (76%) | 9/15 (60%) | 22/27 (81%) | 6/7 (86%) | 4/9 (44%) | 7/10 (70%) | 13/13 (100%) | 12/16 (75%) |
| CRVS | 50/59 (85%) | 12/15 (80%) | 27/32 (84%) | 11/12 (92%) | 8/10 (80%) | 10/12 (83%) | 14/14 (100%) | 17/22 (77%) |
| Wastewater Surveillance | 37/47 (79%) | 9/11 (82%) | 19/26 (73%) | 9/10 (90%) | 6/7 (86%) | 7/7 (100%) | 8/12 (67%) | 15/20 (75%) |
| EBS: Human Health | 50/55 (91%) | 12/13 (92%) | 31/33 (94%) | 7/9 (78%) | 8/8 (100%) | 11/12 (92%) | 10/12 (83%) | 20/22 (91%) |
| EBS: Animal Health | 29/56 (52%) | 9/16 (56%) | 16/30 (53%) | 4/10 (40%) | 4/9 (44%) | 6/10 (60%) | 8/15 (53%) | 10/21 (48%) |
| EBS: Environmental Hazards | 31/52 (60%) | 10/14 (71%) | 18/29 (62%) | 3/9 (33%) | 2/7 (29%) | 7/10 (70%) | 9/13 (69%) | 13/21 (62%) |
| EBS: Biological hazards / biosecurity | 37/53 (70%) | 13/14 (93%) | 18/28 (64%) | 6/11 (55%) | 5/7 (71%) | 9/12 (75%) | 10/12 (83%) | 12/21 (57%) |
| EBS: Food & water security | 42/56 (75%) | 11/13 (85%) | 25/32 (78%) | 6/11 (55%) | 6/8 (75%) | 7/10 (70%) | 11/15 (73%) | 17/22 (77%) |

**Supplementary Table 18. Data collected through surveillance systems are integrated**

| **Data collected through system are integrated** | **Overall** | **IDS Status** | | | **WB Income Group** | | | |
| --- | --- | --- | --- | --- | --- | --- | --- | --- |
| **n/N (%)** |  | **Developed IDS** | **Partial IDS** | **No IDS** | **Low** | **Lower-middle** | **Upper-middle** | **High** |
| Notifiable Diseases | 47/64 (73%) | 15/16 (94%) | 26/36 (72%) | 6/12 (50%) | 7/11 (64%) | 10/14 (71%) | 12/15 (80%) | 17/23 (74%) |
| Syndromic Surveillance | 29/62 (47%) | 8/16 (50%) | 18/34 (53%) | 3/12 (25%) | 4/11 (36%) | 6/15 (40%) | 7/14 (50%) | 11/21 (52%) |
| Sentinel Surveillance | 31/63 (49%) | 9/16 (56%) | 18/35 (51%) | 4/12 (33%) | 5/11 (45%) | 8/13 (62%) | 8/15 (53%) | 9/23 (39%) |
| Lab-based Surveillance | 32/63 (51%) | 13/16 (81%) | 16/35 (46%) | 3/12 (25%) | 5/11 (45%) | 6/13 (46%) | 8/15 (53%) | 12/23 (52%) |
| Hospital-based Surveillance | 19/62 (31%) | 9/15 (60%) | 6/34 (18%) | 4/13 (31%) | 4/11 (36%) | 5/14 (36%) | 4/15 (27%) | 6/22 (27%) |
| Other Facility-based Surveillance | 19/59 (32%) | 10/16 (62%) | 7/31 (23%) | 2/12 (17%) | 3/11 (27%) | 7/13 (54%) | 3/14 (21%) | 5/20 (25%) |
| Case-based Surveillance | 36/61 (59%) | 14/16 (88%) | 17/33 (52%) | 5/12 (42%) | 7/11 (64%) | 7/13 (54%) | 9/14 (64%) | 13/23 (57%) |
| Disease-specific Surveillance | 37/64 (58%) | 12/16 (75%) | 21/36 (58%) | 4/12 (33%) | 7/11 (64%) | 9/14 (64%) | 9/15 (60%) | 11/23 (48%) |
| Community-based Surveillance | 16/46 (35%) | 7/15 (47%) | 5/24 (21%) | 4/7 (57%) | 6/11 (55%) | 5/10 (50%) | 1/10 (10%) | 3/14 (21%) |
| Mortality Surveillance | 22/60 (37%) | 7/14 (50%) | 10/35 (29%) | 5/11 (45%) | 3/9 (33%) | 3/12 (25%) | 7/15 (47%) | 8/23 (35%) |
| Genomic Surveillance | 18/57 (32%) | 5/16 (31%) | 11/30 (37%) | 2/11 (18%) | 2/9 (22%) | 3/13 (23%) | 3/11 (27%) | 9/23 (39%) |
| Behavioral Surveillance | 4/35 (11%) | 3/9 (33%) | 0/21 (0%) | 1/5 (20%) | 0/5 (0%) | 2/6 (33%) | 2/7 (29%) | 0/17 (0%) |
| Vaccination Coverage | 34/64 (53%) | 10/16 (62%) | 19/36 (53%) | 5/12 (42%) | 5/11 (45%) | 9/14 (64%) | 10/15 (67%) | 10/23 (43%) |
| Vaccine Effectiveness | 24/59 (41%) | 7/15 (47%) | 13/33 (39%) | 4/11 (36%) | 4/10 (40%) | 4/12 (33%) | 8/13 (62%) | 8/23 (35%) |
| Survey and Research | 6/49 (12%) | 5/15 (33%) | 1/27 (3.7%) | 0/7 (0%) | 2/9 (22%) | 2/10 (20%) | 1/13 (7.7%) | 1/16 (6.2%) |
| CRVS | 16/59 (27%) | 6/15 (40%) | 5/32 (16%) | 5/12 (42%) | 4/10 (40%) | 2/12 (17%) | 3/14 (21%) | 6/22 (27%) |
| Wastewater Surveillance | 11/47 (23%) | 6/11 (55%) | 5/26 (19%) | 0/10 (0%) | 1/7 (14%) | 3/7 (43%) | 2/12 (17%) | 5/20 (25%) |
| EBS: Human Health | 22/55 (40%) | 8/13 (62%) | 12/33 (36%) | 2/9 (22%) | 4/8 (50%) | 6/12 (50%) | 3/12 (25%) | 9/22 (41%) |
| EBS: Animal Health | 11/56 (20%) | 7/16 (44%) | 3/30 (10%) | 1/10 (10%) | 2/9 (22%) | 3/10 (30%) | 2/15 (13%) | 3/21 (14%) |
| EBS: Environmental Hazards | 10/52 (19%) | 6/14 (43%) | 3/29 (10%) | 1/9 (11%) | 1/7 (14%) | 3/10 (30%) | 2/13 (15%) | 4/21 (19%) |
| EBS: Biological hazards / biosecurity | 12/53 (23%) | 7/14 (50%) | 4/28 (14%) | 1/11 (9.1%) | 2/7 (29%) | 3/12 (25%) | 2/12 (17%) | 4/21 (19%) |
| EBS: Food & water security | 11/56 (20%) | 6/13 (46%) | 3/32 (9.4%) | 2/11 (18%) | 1/8 (12%) | 4/10 (40%) | 2/15 (13%) | 3/22 (14%) |

**Supplementary Table 19. Specific surveillance systems or data sources from the sectors contributing to surveillance system that cannot be integrated**

| **Specific surveillance systems or data sources that cannot be integrated** | **Overall** | **IDS Status** | | **WB Income Group** | | | |
| --- | --- | --- | --- | --- | --- | --- | --- |
| **n/N (%)** |  | **Developed IDS** | **Partial IDS** | **Low** | **Lower-middle** | **Upper-middle** | **High** |
| Public Sector Providers | 10/51 (20%) | 2/16 (13%) | 8/35 (23%) | 0/9 (0%) | 2/11 (18%) | 2/13 (15%) | 6/18 (33%) |
| Private Sector Providers | 24/51 (47%) | 4/16 (25%) | 20/35 (57%) | 5/9 (56%) | 4/11 (36%) | 7/13 (54%) | 8/18 (44%) |
| Public Health Sector | 10/51 (20%) | 1/16 (6%) | 9/35 (26%) | 2/9 (22%) | 3/11 (27%) | 2/13 (15%) | 3/18 (17%) |
| Animal Health Sector | 31/51 (61%) | 7/16 (44%) | 24/35 (69%) | 7/9 (78%) | 9/11 (82%) | 6/13 (46%) | 9/18 (50%) |
| Agricultural Sector | 27/51 (53%) | 5/16 (31%) | 22/35 (63%) | 7/9 (78%) | 5/11 (45%) | 6/13 (46%) | 9/18 (50%) |
| Environmental Sector | 26/51 (51%) | 5/16 (31%) | 21/35 (60%) | 6/9 (67%) | 5/11 (45%) | 6/13 (46%) | 9/18 (50%) |
| Laboratories | 14/51 (27%) | 2/16 (13%) | 12/35 (34%) | 3/9 (33%) | 4/11 (36%) | 1/13 (8%) | 6/18 (33%) |
| Private Sector | 25/51 (49%) | 5/16 (31%) | 20/35 (57%) | 5/9 (56%) | 6/11 (55%) | 7/13 (54%) | 7/18 (39%) |
| Communities | 19/51 (37%) | 4/16 (25%) | 15/35 (43%) | 2/9 (22%) | 5/11 (45%) | 6/13 (46%) | 6/18 (33%) |
| Pharmaceutical Sector | 28/51 (55%) | 6/16 (38%) | 22/35 (63%) | 5/9 (56%) | 6/11 (55%) | 7/13 (54%) | 10/18 (56%) |
| Occupational Health | 24/51 (47%) | 6/16 (38%) | 18/35 (51%) | 4/9 (44%) | 5/11 (45%) | 5/13 (38%) | 10/18 (56%) |
| Chemical and Poison Sector | 25/51 (49%) | 7/16 (44%) | 18/35 (51%) | 5/9 (56%) | 7/11 (64%) | 6/13 (46%) | 7/18 (39%) |
| Disaster Management | 23/51 (45%) | 5/16 (31%) | 18/35 (51%) | 6/9 (67%) | 6/11 (55%) | 4/13 (31%) | 7/18 (39%) |
| Food Safety | 24/51 (47%) | 5/16 (31%) | 19/35 (54%) | 5/9 (56%) | 7/11 (64%) | 5/13 (38%) | 7/18 (39%) |
| Biosafety and Biosecurity | 21/51 (41%) | 4/16 (25%) | 17/35 (49%) | 5/9 (56%) | 6/11 (55%) | 4/13 (31%) | 6/18 (33%) |

**Supplementary Table 20. Disease-specific programs that are not integrated**

| **Disease-specific programs that are not integrated** | **Overall** | **IDS Status** | | **WB Income Group** | | | |
| --- | --- | --- | --- | --- | --- | --- | --- |
| **n/N (%)** |  | **Developed IDS** | **Partial IDS** | **Low** | **Lower-middle** | **Upper-middle** | **High** |
| HIV/AIDS | 13/51 (25%) | 7/16 (44%) | 6/35 (17%) | 2/9 (22%) | 3/11 (27%) | 4/13 (31%) | 4/18 (22%) |
| Malaria | 8/51 (16%) | 4/16 (25%) | 4/35 (11%) | 2/9 (22%) | 2/11 (18%) | 1/13 (8%) | 3/18 (17%) |
| TB | 9/51 (18%) | 4/16 (25%) | 5/35 (14%) | 2/9 (22%) | 1/11 (9%) | 2/13 (15%) | 4/18 (22%) |
| Cholera | 5/51 (10%) | 1/16 (6%) | 4/35 (11%) | 2/9 (22%) | 0/11 (0%) | 1/13 (8%) | 2/18 (11%) |
| Measles | 6/51 (12%) | 2/16 (13%) | 4/35 (11%) | 2/9 (22%) | 0/11 (0%) | 2/13 (15%) | 2/18 (11%) |
| COVID-19 | 8/51 (16%) | 3/16 (19%) | 5/35 (14%) | 3/9 (33%) | 1/11 (9%) | 2/13 (15%) | 2/18 (11%) |
| Non-communicable diseases (NCDs) | 26/51 (51%) | 6/16 (38%) | 20/35 (57%) | 1/9 (11%) | 5/11 (45%) | 7/13 (54%) | 13/18 (72%) |

**Supplementary Table 21. Current surveillance system allows for integration of surveillance data for public health response**

| **System allows for integration** | **Overall** | **IDS Status** | | | **WB Income Group** | | | |
| --- | --- | --- | --- | --- | --- | --- | --- | --- |
| **n/N (%)** |  | **Developed IDS** | **Partial IDS** | **No IDS** | **Low** | **Lower-middle** | **Upper-middle** | **High** |
| Yes | 43/65 (66%) | 10/16 (62%) | 28/36 (78%) | 5/13 (38%) | 7/11 (64%) | 11/15 (73%) | 10/15 (67%) | 14/23 (61%) |
| No | 12/65 (18%) | 4/16 (25%) | 2/36 (5.6%) | 6/13 (46%) | 4/11 (36%) | 3/15 (20%) | 2/15 (13%) | 3/23 (13%) |
| Other (partial) | 10/65 (15%) | 2/16 (12%) | 6/36 (17%) | 2/13 (15%) | 0/11 (0%) | 1/15 (6.7%) | 3/15 (20%) | 6/23 (26%) |

**Supplementary Table 22. Lab data reported to IDS system**

| **Lab data reported to IDS System** | **Overall** | **IDS Status** | | **WB Income Group** | | | |
| --- | --- | --- | --- | --- | --- | --- | --- |
| **n/N (%)** |  | **Developed IDS** | **Partial IDS** | **Low** | **Lower-middle** | **Upper-middle** | **High** |
| National | 51/52 (98%) | 16/16 (100%) | 35/36 (97%) | 9/9 (100%) | 11/11 (100%) | 13/13 (100%) | 17/18 (94%) |
| Subnational | 37/52 (71%) | 12/16 (75%) | 25/36 (69%) | 7/9 (78%) | 8/11 (73%) | 11/13 (85%) | 11/18 (61%) |
| Other public sector | 34/52 (65%) | 12/16 (75%) | 22/36 (61%) | 6/9 (67%) | 7/11 (64%) | 9/13 (69%) | 12/18 (67%) |
| Private | 32/52 (62%) | 10/16 (63%) | 22/36 (61%) | 4/9 (44%) | 5/11 (45%) | 9/13 (69%) | 14/18 (78%) |
| Regional | 21/52 (40%) | 5/16 (31%) | 16/36 (44%) | 3/9 (33%) | 6/11 (55%) | 5/13 (38%) | 7/18 (39%) |

**Supplementary Table 23. Mechanism for integrating lab data into IDS system**

| **Mechanism for integrating lab data into IDS system** | **Overall** | **IDS Status** | | **WB Income Group** | | | |
| --- | --- | --- | --- | --- | --- | --- | --- |
| **n/N (%)** |  | **Developed IDS** | **Partial IDS** | **Low** | **Lower-middle** | **Upper-middle** | **High** |
| IT | 35/50 (70%) | 9/16 (56%) | 26/34 (76%) | 6/9 (67%) | 4/11 (36%) | 10/13 (77%) | 15/17 (88%) |
| Other electronic | 15/50 (30%) | 4/16 (25%) | 11/34 (32%) | 2/9 (22%) | 4/11 (36%) | 3/13 (23%) | 6/17 (35%) |
| Manual | 15/50 (30%) | 6/16 (38%) | 9/34 (26%) | 3/9 (33%) | 3/11 (27%) | 4/13 (31%) | 5/17 (29%) |
| Not integrated into IDS system | 8/50 (16%) | 2/16 (13%) | 6/34 (18%) | 2/9 (22%) | 4/11 (36%) | 1/13 (8%) | 1/17 (6%) |

**Supplementary Table 24. Genomic testing**

| **Genomic testing availability** | **Overall** | **IDS Status** | | **WB Income Group** | | | |
| --- | --- | --- | --- | --- | --- | --- | --- |
| **n/N (%)** |  | **Developed IDS** | **Partial IDS** | **Low** | **Lower-middle** | **Upper-middle** | **High** |
| Available from NPHLs | 39/49 (80%) | 12/15 (80%) | 27/34 (79%) | 5/9 (56%) | 9/11 (82%) | 8/12 (67%) | 17/17 (100%) |
| Available from subnational labs | 14/49 (29%) | 3/15 (20%) | 11/34 (32%) | 1/9 (11%) | 0/11 (0%) | 1/12 (8%) | 12/17 (71%) |
| Available from private labs | 11/49 (22%) | 2/15 (13%) | 9/34 (26%) | 0/9 (0%) | 1/11 (9%) | 1/12 (8%) | 9/17 (53%) |
| Available outside of the country | 10/49 (20%) | 2/15 (13%) | 8/34 (24%) | 1/9 (11%) | 3/11 (27%) | 3/12 (25%) | 3/17 (18%) |
| Not available | 6/49 (12%) | 2/15 (13%) | 4/34 (12%) | 3/9 (33%) | 1/11 (9%) | 2/12 (17%) | 0/17 (0%) |

**Supplementary Table 25. Challenges experienced in setting up IDS system**

| **Challenges experienced in setting up your IDS system** | **Overall** | **IDS Status** | | **WB Income Group** | | | |
| --- | --- | --- | --- | --- | --- | --- | --- |
| **n/N (%)** |  | **Developed IDS** | **Partial IDS** | **Low** | **Lower-middle** | **Upper-middle** | **High** |
| Governance | 31/50 (62%) | 7/16 (44%) | 24/34 (71%) | 5/9 (56%) | 8/11 (73%) | 6/13 (46%) | 12/17 (71%) |
| Financial | 37/50 (74%) | 12/16 (75%) | 25/34 (74%) | 8/9 (89%) | 10/11 (91%) | 7/13 (54%) | 12/17 (71%) |
| Data availability | 30/50 (60%) | 9/16 (56%) | 21/34 (62%) | 7/9 (78%) | 7/11 (64%) | 6/13 (46%) | 10/17 (59%) |
| Data sharing and ownership | 33/50 (66%) | 8/16 (50%) | 25/34 (74%) | 3/9 (33%) | 10/11 (91%) | 6/13 (46%) | 14/17 (82%) |
| IT systems interoperability | 42/50 (84%) | 13/16 (81%) | 29/34 (85%) | 7/9 (78%) | 11/11 (100%) | 9/13 (69%) | 15/17 (88%) |
| Analysis and reporting | 27/50 (54%) | 9/16 (56%) | 18/34 (53%) | 6/9 (67%) | 9/11 (82%) | 7/13 (54%) | 5/17 (29%) |
| Laboratory | 23/50 (46%) | 10/16 (63%) | 13/34 (38%) | 6/9 (67%) | 8/11 (73%) | 5/13 (38%) | 4/17 (24%) |
| Workforce capacity and capabilities | 30/50 (60%) | 8/16 (50%) | 22/34 (65%) | 5/9 (56%) | 9/11 (82%) | 6/13 (46%) | 10/17 (59%) |

**Supplementary Table 26. Barriers to IDS in settings without IDS**

| **Barriers to IDS** | **No IDS** | **WB Income Group** | | | |
| --- | --- | --- | --- | --- | --- |
| **n/N (%)** |  | **Low** | **Lower-middle** | **Upper-middle** | **High** |
| Governance | 10/13 (77%) | 1/2 (50%) | 3/4 (75%) | 2/2 (100%) | 4/5 (80%) |
| Financial | 7/13 (54%) | 1/2 (50%) | 1/4 (25%) | 2/2 (100%) | 3/5 (60%) |
| Data availability | 8/13 (62%) | 0/2 (0%) | 4/4 (100%) | 1/2 (50%) | 3/5 (60%) |
| Data sharing and ownership | 10/13 (77%) | 1/2 (50%) | 4/4 (100%) | 1/2 (50%) | 4/5 (80%) |
| IT systems interoperability | 10/13 (77%) | 1/2 (50%) | 3/4 (75%) | 2/2 (100%) | 4/5 (80%) |
| Analysis and reporting | 2/13 (15%) | 0/2 (0%) | 1/4 (25%) | 0/2 (0%) | 1/5 (20%) |
| Laboratory | 2/13 (15%) | 0/2 (0%) | 0/4 (0%) | 0/2 (0%) | 2/5 (40%) |
| Workforce capacity and capabilities | 8/13 (62%) | 0/2 (0%) | 3/4 (75%) | 1/2 (50%) | 4/5 (80%) |

**Supplementary Table 27. Priorities in development of the surveillance system**

| **Priorities in development of the surveillance system** | **Overall** | **IDS Status** | | | **WB Income Group** | | | |
| --- | --- | --- | --- | --- | --- | --- | --- | --- |
| **n/N (%)** |  | **Developed IDS** | **Partial IDS** | **No IDS** | **Low** | **Lower-middle** | **Upper-middle** | **High** |
| Greater cross-border sharing of surveillance data with other countries and across the region |  |  |  |  |  |  |  |  |
| Not a priority | 9/62 (15%) | 2/16 (12%) | 3/34 (8.8%) | 4/12 (33%) | 1/11 (9.1%) | 0/15 (0%) | 3/15 (20%) | 5/20 (25%) |
| Priority | 35/62 (56%) | 10/16 (62%) | 19/34 (56%) | 6/12 (50%) | 5/11 (45%) | 10/15 (67%) | 9/15 (60%) | 11/20 (55%) |
| High priority | 18/62 (29%) | 4/16 (25%) | 12/34 (35%) | 2/12 (17%) | 5/11 (45%) | 5/15 (33%) | 3/15 (20%) | 4/20 (20%) |
| Greater integration of surveillance systems at the national level |  |  |  |  |  |  |  |  |
| Not a priority | 2/63 (3.2%) | 1/16 (6.2%) | 0/34 (0%) | 1/13 (7.7%) | 1/11 (9.1%) | 0/15 (0%) | 0/15 (0%) | 1/21 (4.8%) |
| Priority | 15/63 (24%) | 5/16 (31%) | 8/34 (24%) | 2/13 (15%) | 1/11 (9.1%) | 4/15 (27%) | 4/15 (27%) | 6/21 (29%) |
| High priority | 46/63 (73%) | 10/16 (62%) | 26/34 (76%) | 10/13 (77%) | 9/11 (82%) | 11/15 (73%) | 11/15 (73%) | 14/21 (67%) |
| Greater integration of surveillance systems at the sub-national level |  |  |  |  |  |  |  |  |
| Not a priority | 6/62 (9.7%) | 2/16 (12%) | 3/33 (9.1%) | 1/13 (7.7%) | 1/11 (9.1%) | 0/15 (0%) | 0/15 (0%) | 5/20 (25%) |
| Priority | 17/62 (27%) | 4/16 (25%) | 10/33 (30%) | 3/13 (23%) | 3/11 (27%) | 5/15 (33%) | 4/15 (27%) | 5/20 (25%) |
| High priority | 39/62 (63%) | 10/16 (62%) | 20/33 (61%) | 9/13 (69%) | 7/11 (64%) | 10/15 (67%) | 11/15 (73%) | 10/20 (50%) |
| Training and development of the workforce to support surveillance |  |  |  |  |  |  |  |  |
| Not a priority | 5/63 (7.9%) | 2/16 (12%) | 1/34 (2.9%) | 2/13 (15%) | 0/11 (0%) | 0/15 (0%) | 0/15 (0%) | 5/21 (24%) |
| Priority | 19/63 (30%) | 3/16 (19%) | 13/34 (38%) | 3/13 (23%) | 1/11 (9.1%) | 5/15 (33%) | 4/15 (27%) | 9/21 (43%) |
| High priority | 39/63 (62%) | 11/16 (69%) | 20/34 (59%) | 8/13 (62%) | 10/11 (91%) | 10/15 (67%) | 11/15 (73%) | 7/21 (33%) |
| Development of technical guidelines for integration of surveillance data |  |  |  |  |  |  |  |  |
| Not a priority | 4/63 (6.3%) | 2/16 (12%) | 2/34 (5.9%) | 0/13 (0%) | 0/11 (0%) | 0/15 (0%) | 0/15 (0%) | 4/21 (19%) |
| Priority | 31/63 (49%) | 8/16 (50%) | 18/34 (53%) | 5/13 (38%) | 4/11 (36%) | 8/15 (53%) | 8/15 (53%) | 11/21 (52%) |
| High priority | 28/63 (44%) | 6/16 (38%) | 14/34 (41%) | 8/13 (62%) | 7/11 (64%) | 7/15 (47%) | 7/15 (47%) | 6/21 (29%) |
| Monitoring and evaluation of surveillance systems to improve performance |  |  |  |  |  |  |  |  |
| Not a priority | 7/63 (11%) | 2/16 (12%) | 3/34 (8.8%) | 2/13 (15%) | 0/11 (0%) | 0/15 (0%) | 1/15 (6.7%) | 6/21 (29%) |
| Priority | 30/63 (48%) | 8/16 (50%) | 18/34 (53%) | 4/13 (31%) | 4/11 (36%) | 9/15 (60%) | 7/15 (47%) | 10/21 (48%) |
| High priority | 26/63 (41%) | 6/16 (38%) | 13/34 (38%) | 7/13 (54%) | 7/11 (64%) | 6/15 (40%) | 7/15 (47%) | 5/21 (24%) |
| Situational analysis to identify gaps in the surveillance system |  |  |  |  |  |  |  |  |
| Not a priority | 3/62 (4.8%) | 0/16 (0%) | 1/33 (3.0%) | 2/13 (15%) | 0/11 (0%) | 0/14 (0%) | 1/15 (6.7%) | 2/21 (9.5%) |
| Priority | 34/62 (55%) | 10/16 (62%) | 20/33 (61%) | 4/13 (31%) | 4/11 (36%) | 7/14 (50%) | 10/15 (67%) | 13/21 (62%) |
| High priority | 25/62 (40%) | 6/16 (38%) | 12/33 (36%) | 7/13 (54%) | 7/11 (64%) | 7/14 (50%) | 4/15 (27%) | 6/21 (29%) |
| Securing access to data collected by public sector / governmental organisations that is not currently collected by NPHI / the relevant public health authority |  |  |  |  |  |  |  |  |
| Not a priority | 5/63 (7.9%) | 2/16 (12%) | 2/34 (5.9%) | 1/13 (7.7%) | 0/11 (0%) | 2/15 (13%) | 0/15 (0%) | 3/21 (14%) |
| Priority | 37/63 (59%) | 9/16 (56%) | 19/34 (56%) | 9/13 (69%) | 6/11 (55%) | 8/15 (53%) | 12/15 (80%) | 11/21 (52%) |
| High priority | 21/63 (33%) | 5/16 (31%) | 13/34 (38%) | 3/13 (23%) | 5/11 (45%) | 5/15 (33%) | 3/15 (20%) | 7/21 (33%) |
| Securing access to data collected by private sector and non-governmental organisations that is not currently collected by NPHI / the relevant public health authority |  |  |  |  |  |  |  |  |
| Not a priority | 13/63 (21%) | 5/16 (31%) | 4/34 (12%) | 4/13 (31%) | 2/11 (18%) | 2/15 (13%) | 1/15 (6.7%) | 7/21 (33%) |
| Priority | 34/63 (54%) | 7/16 (44%) | 19/34 (56%) | 8/13 (62%) | 5/11 (45%) | 9/15 (60%) | 10/15 (67%) | 10/21 (48%) |
| High priority | 16/63 (25%) | 4/16 (25%) | 11/34 (32%) | 1/13 (7.7%) | 4/11 (36%) | 4/15 (27%) | 4/15 (27%) | 4/21 (19%) |
| Securing legislative /legal mandate to collect surveillance data needed for planning, preparedness and response work |  |  |  |  |  |  |  |  |
| Not a priority | 10/63 (16%) | 4/16 (25%) | 2/34 (5.9%) | 4/13 (31%) | 3/11 (27%) | 3/15 (20%) | 1/15 (6.7%) | 2/21 (9.5%) |
| Priority | 23/63 (37%) | 4/16 (25%) | 16/34 (47%) | 3/13 (23%) | 2/11 (18%) | 4/15 (27%) | 8/15 (53%) | 9/21 (43%) |
| High priority | 30/63 (48%) | 8/16 (50%) | 16/34 (47%) | 6/13 (46%) | 6/11 (55%) | 8/15 (53%) | 6/15 (40%) | 10/21 (48%) |
| Developing timely and transparent reporting systems for the surveillance data that is collected |  |  |  |  |  |  |  |  |
| Not a priority | 3/63 (4.8%) | 1/16 (6.2%) | 0/34 (0%) | 2/13 (15%) | 0/11 (0%) | 0/15 (0%) | 1/15 (6.7%) | 2/21 (9.5%) |
| Priority | 28/63 (44%) | 7/16 (44%) | 17/34 (50%) | 4/13 (31%) | 4/11 (36%) | 7/15 (47%) | 7/15 (47%) | 10/21 (48%) |
| High priority | 32/63 (51%) | 8/16 (50%) | 17/34 (50%) | 7/13 (54%) | 7/11 (64%) | 8/15 (53%) | 7/15 (47%) | 9/21 (43%) |
| Securing political support for the further development of surveillance systems |  |  |  |  |  |  |  |  |
| Not a priority | 11/63 (17%) | 3/16 (19%) | 4/34 (12%) | 4/13 (31%) | 1/11 (9.1%) | 1/15 (6.7%) | 1/15 (6.7%) | 8/21 (38%) |
| Priority | 29/63 (46%) | 8/16 (50%) | 16/34 (47%) | 5/13 (38%) | 8/11 (73%) | 5/15 (33%) | 8/15 (53%) | 8/21 (38%) |
| High priority | 23/63 (37%) | 5/16 (31%) | 14/34 (41%) | 4/13 (31%) | 2/11 (18%) | 9/15 (60%) | 6/15 (40%) | 5/21 (24%) |
| Development of the requisite IT and digital infrastructure and tools needed to support surveillance systems |  |  |  |  |  |  |  |  |
| Not a priority | 4/63 (6.3%) | 1/16 (6.2%) | 2/34 (5.9%) | 1/13 (7.7%) | 1/11 (9.1%) | 0/15 (0%) | 0/15 (0%) | 3/21 (14%) |
| Priority | 21/63 (33%) | 8/16 (50%) | 10/34 (29%) | 3/13 (23%) | 2/11 (18%) | 6/15 (40%) | 6/15 (40%) | 7/21 (33%) |
| High priority | 38/63 (60%) | 7/16 (44%) | 22/34 (65%) | 9/13 (69%) | 8/11 (73%) | 9/15 (60%) | 9/15 (60%) | 11/21 (52%) |
| Increasing interconnectedness and interoperability of surveillance systems |  |  |  |  |  |  |  |  |
| Not a priority | 4/63 (6.3%) | 2/16 (12%) | 2/34 (5.9%) | 0/13 (0%) | 0/11 (0%) | 1/15 (6.7%) | 0/15 (0%) | 3/21 (14%) |
| Priority | 21/63 (33%) | 6/16 (38%) | 10/34 (29%) | 5/13 (38%) | 5/11 (45%) | 3/15 (20%) | 7/15 (47%) | 6/21 (29%) |
| High priority | 38/63 (60%) | 8/16 (50%) | 22/34 (65%) | 8/13 (62%) | 6/11 (55%) | 11/15 (73%) | 8/15 (53%) | 12/21 (57%) |
| Increasing access to and capacity of laboratory services to support surveillance systems |  |  |  |  |  |  |  |  |
| Not a priority | 2/63 (3.2%) | 0/16 (0%) | 2/34 (5.9%) | 0/13 (0%) | 0/11 (0%) | 0/15 (0%) | 0/15 (0%) | 2/21 (9.5%) |
| Priority | 30/63 (48%) | 10/16 (62%) | 14/34 (41%) | 6/13 (46%) | 4/11 (36%) | 7/15 (47%) | 8/15 (53%) | 11/21 (52%) |
| High priority | 31/63 (49%) | 6/16 (38%) | 18/34 (53%) | 7/13 (54%) | 7/11 (64%) | 8/15 (53%) | 7/15 (47%) | 8/21 (38%) |
| Improving access to genomic surveillance tools |  |  |  |  |  |  |  |  |
| Not a priority | 6/63 (9.5%) | 2/16 (12%) | 4/34 (12%) | 0/13 (0%) | 0/11 (0%) | 2/15 (13%) | 1/15 (6.7%) | 3/21 (14%) |
| Priority | 34/63 (54%) | 9/16 (56%) | 16/34 (47%) | 9/13 (69%) | 6/11 (55%) | 8/15 (53%) | 10/15 (67%) | 10/21 (48%) |
| High priority | 23/63 (37%) | 5/16 (31%) | 14/34 (41%) | 4/13 (31%) | 5/11 (45%) | 5/15 (33%) | 4/15 (27%) | 8/21 (38%) |
| Improving data transparency, to ensure the visibility of all national threats by NPHIs and by WHO for transnational threats |  |  |  |  |  |  |  |  |
| Not a priority | 8/62 (13%) | 2/15 (13%) | 4/34 (12%) | 2/13 (15%) | 1/10 (10%) | 1/15 (6.7%) | 1/15 (6.7%) | 5/21 (24%) |
| Priority | 28/62 (45%) | 4/15 (27%) | 17/34 (50%) | 7/13 (54%) | 4/10 (40%) | 4/15 (27%) | 9/15 (60%) | 11/21 (52%) |
| High priority | 26/62 (42%) | 9/15 (60%) | 13/34 (38%) | 4/13 (31%) | 5/10 (50%) | 10/15 (67%) | 5/15 (33%) | 5/21 (24%) |
| Securing funding for the development of surveillance systems |  |  |  |  |  |  |  |  |
| Not a priority | 7/63 (11%) | 2/16 (12%) | 3/34 (8.8%) | 2/13 (15%) | 0/11 (0%) | 0/15 (0%) | 0/15 (0%) | 7/21 (33%) |
| Priority | 22/63 (35%) | 5/16 (31%) | 12/34 (35%) | 5/13 (38%) | 1/11 (9.1%) | 6/15 (40%) | 7/15 (47%) | 8/21 (38%) |
| High priority | 34/63 (54%) | 9/16 (56%) | 19/34 (56%) | 6/13 (46%) | 10/11 (91%) | 9/15 (60%) | 8/15 (53%) | 6/21 (29%) |

**Supplementary Table 28. IDS financing**

| **IDS Financing** | **Overall** | **IDS Status** | | **WB Income Group** | | | |
| --- | --- | --- | --- | --- | --- | --- | --- |
| **n/N (%)** |  | **Developed IDS** | **Partial IDS** | **Low** | **Lower-middle** | **Upper-middle** | **High** |
| National government | 37/48 (77%) | 15/15 (100%) | 22/33 (67%) | 8/9 (89%) | 8/11 (73%) | 8/11 (73%) | 13/17 (76%) |
| NGO/Philanthropic | 7/48 (15%) | 5/15 (33%) | 2/33 (6%) | 3/9 (33%) | 4/11 (36%) | 0/11 (0%) | 0/17 (0%) |
| Private | 1/48 (2%) | 0/15 (0%) | 1/33 (3%) | 0/9 (0%) | 1/11 (9%) | 0/11 (0%) | 0/17 (0%) |
| International aid funding from other country partner | 14/48 (29%) | 7/15 (47%) | 6/33 (18%) | 5/9 (56%) | 6/11 (55%) | 1/11 (9%) | 2/17 (12%) |
| International aid funding from a non-government organization | 14/48 (29%) | 7/15 (47%) | 4/33 (12%) | 6/9 (67%) | 7/11 (64%) | 0/11 (0%) | 1/17 (6%) |

**Supplementary Table 29. Extent that integration of the different areas of surveillance system is supported by ‘semantic consistency’**

| **Integration level** | **Overall** | **IDS Status** | | | **WB Income Group** | | | |
| --- | --- | --- | --- | --- | --- | --- | --- | --- |
| **n/N (%)** |  | **Developed IDS** | **Partial IDS** | **No IDS** | **Low** | **Lower-middle** | **Upper-middle** | **High** |
| Not at all | 4/63 (6%) | 1/16 (6%) | 3/35 (9%) | 0/12 (0%) | 0/10 (0%) | 1/15 (7%) | 1/15 (7%) | 2/22 (9%) |
| To some extent | 42/63 (67%) | 9/16 (56%) | 25/35 (71%) | 8/12 (67%) | 4/10 (40%) | 13/15 (87%) | 9/15 (60%) | 16/22 (73%) |
| To a great extent | 17/63 (27%) | 6/16 (38%) | 7/35 (20%) | 4/12 (33%) | 6/10 (60%) | 1/15 (7%) | 5/15 (33%) | 4/22 (18%) |

Level of integration:

- Not at all – different surveillance systems use different languages, data definitions, and formats. These are a barrier to greater integration.
- To some extent – a number of surveillance systems operate use standardised language, data definitions, and formats to support their integration. However, this is not the case across all surveillance systems, and there is more work to do in this regard.
- To a great extent – almost all surveillance systems operate using a standardised language, data definitions, and formats which supports integration across the system.

**Supplementary Table 30. Barriers to lab integration**

| **Barriers to lab data integration** | **Overall** | **IDS Status** | | **WB Income Group** | | | |
| --- | --- | --- | --- | --- | --- | --- | --- |
| **n/N (%)** |  | **Developed IDS** | **Partial IDS** | **Low** | **Lower-middle** | **Upper-middle** | **High** |
| Limited staff | 20/50 (40%) | 6/16 (38%) | 14/34 (41%) | 5/9 (56%) | 7/11 (64%) | 5/13 (38%) | 3/17 (18%) |
| Lack of equipment/supplies | 20/50 (40%) | 7/16 (44%) | 13/34 (38%) | 6/9 (67%) | 8/11 (73%) | 4/13 (31%) | 2/17 (12%) |
| Inefficient specimen transfer | 9/50 (18%) | 4/16 (25%) | 5/34 (15%) | 3/9 (33%) | 5/11 (45%) | 0/13 (0%) | 1/17 (6%) |
| Poor data systems/integration | 27/50 (54%) | 8/16 (50%) | 19/34 (56%) | 4/9 (44%) | 9/11 (82%) | 8/13 (62%) | 6/17 (35%) |
| No barriers to report | 10/50 (20%) | 4/16 (25%) | 6/34 (18%) | 1/9 (11%) | 0/11 (0%) | 3/13 (23%) | 6/17 (35%) |
